# Differentiation by nerve growth factor (NGF) involves mechanisms of crosstalk between energy homeostasis and mitochondrial remodeling

**DOI:** 10.1038/s41419-018-0429-9

**Published:** 2018-03-09

**Authors:** Francesca Martorana, Daniela Gaglio, Maria Rosaria Bianco, Federica Aprea, Assunta Virtuoso, Marcella Bonanomi, Lilia Alberghina, Michele Papa, Anna Maria Colangelo

**Affiliations:** 1 Laboratory of Neuroscience “R. Levi-Montalcini”, Department of Biotechnology and Biosciences, University of Milano-Bicocca, 20126 Milano, Italy; 2 SYSBIO.IT, Centre of Systems Biology, University of Milano-Bicocca, Milano, Italy; 3 Institute of Molecular Bioimaging and Physiology, National Research Council (IBFM-CNR), Segrate, MI, Italy; 4 Laboratory of Morphology of Neuronal Network, Department of Public Medicine, University of Campania “Luigi Vanvitelli”, Napoli, Italy; 5 NeuroMI Milan Center for Neuroscience, University of Milano-Bicocca, Milano, Italy

## Abstract

Neuronal differentiation involves extensive modification of biochemical and morphological properties to meet novel functional requirements. Reorganization of the mitochondrial network to match the higher energy demand plays a pivotal role in this process. Mechanisms of neuronal differentiation in response to nerve growth factor (NGF) have been largely characterized in terms of signaling, however, little is known about its impact on mitochondrial remodeling and metabolic function. In this work, we show that NGF-induced differentiation requires the activation of autophagy mediated by Atg9b and Ambra1, as it is disrupted by their genetic knockdown and by autophagy blockers. NGF differentiation involves the induction of P-AMPK and P-CaMK, and is prevented by their pharmacological inhibition. These molecular events correlate with modifications of energy and redox homeostasis, as determined by ATP and NADPH changes, higher oxygen consumption (OCR) and ROS production. Our data indicate that autophagy aims to clear out exhausted mitochondria, as determined by enhanced localization of p62 and Lysotracker-red to mitochondria. In addition, we newly demonstrate that NGF differentiation is accompanied by increased mitochondrial remodeling involving higher levels of fission (P-Drp1) and fusion proteins (Opa1 and Mfn2), as well as induction of Sirt3 and the transcription factors mtTFA and PPARγ, which regulate mitochondria biogenesis and metabolism to sustain increased mitochondrial mass, potential, and bioenergetics. Overall, our data indicate a new NGF-dependent mechanism involving mitophagy and extensive mitochondrial remodeling, which plays a key role in both neurogenesis and nerve regeneration.

## Introduction

Cell differentiation is a complex process that requires modifications of biochemical and morphological properties to meet novel specialized functions. Neuronal differentiation, in particular, involves extensive remodeling of mitochondria and their distribution along newly formed neurite processes^1,2^.

Nerve growth factor (NGF) is crucial for differentiation and maintenance of specific neuronal populations^3,4^ through activation of the tyrosine kinase TrkA and the p75 receptors, and their well-characterized signaling^5^. Specifically, axonal growth also involves localized increase of intracellular Ca^2+^ (refs. ^6,7^), trafficking of mitochondria to the axonal branches^2,8^ and increased mitochondrial membrane potential^9,10^, suggesting the relevance of mitochondria in sustaining growth cone activity in response to NGF.

Mitochondria play a crucial role during neurogenesis and in post-mitotic neurons by supplying the energy requested for growth cone activity, axonal growth, and synaptic function^11^. Several studies found that neuronal differentiation is accompanied by metabolic reprogramming to meet the increased energy demand. This is achieved by fostering glucose and glutamine metabolism^12,13^, as well as the oxidative phosphorylation^14,15^, thus leading to higher generation of ROS and the need to increase mitochondrial biogenesis^12^ and quality control by mitophagy^13^.

Increasing evidence accumulated about the role of autophagy in differentiation and development^16^. Autophagy was found to regulate the differentiation of neural stem cells^17^, neuroblastoma^18^, retinal ganglion cells^13^, and myoblasts^19,20^. During autophagy, damaged proteins and/or organelles are sequestered within autophagosomes through a complex process regulated by autophagy-related (Atg) proteins. Autophagosomes fuse with lysosomes for degradation of their content, and the breakdown products are recycled as building blocks to maintain metabolic homeostasis under stress conditions^21,22^. In addition to Atg proteins, autophagy during neurogenesis was found to be regulated by Ambra1 (activating molecule in Beclin-1-regulated autophagy), whose deficiency caused neural tube defect^23,24^.

Autophagy during differentiation of myoblasts and neuroblastoma resulted to be induced by AMP-activated kinase (AMPK)^18,19^, a sensor of energy metabolism that activates autophagy through inhibition of mammalian TOR (mTOR)^22,25^. Phospho(Thr172)-AMPK can be induced by a rise in cellular AMP:ATP ratio and by reactive oxygen species (ROS)^22,25^, as well as by Ca^2+^-calmodulin-dependent protein kinase (CaMKK)^26,27^. In myoblasts and retinal ganglion cells, autophagy involved the selective removal of mitochondria^13,20^.

Mitochondrial dynamics is crucial during axonal growth. Mitochondrial biogenesis^12^ and cycles of fission–fusion regulate mitochondrial transition between elongated and fragmented mitochondria for translocation to neurites or removal by mitophagy^28,29^. Fragmentation is controlled by dynamin-related protein-1 (Drp1) through PKA or CaMKI phosphorylation, whereas optic atrophy-1 (Opa1) and mitofusin-1/2 (Mfn1-2) regulate mitochondrial fusion from the inner and outer mitochondrial membrane, respectively^28–31^.

In this study, we demonstrate that NGF-induced differentiation involves modulation of Atg9-Ambra1-dependent mitophagy through activation of P-AMPK and P-CaMK triggered by altered energy homeostasis and mobilization of intracellular Ca^2+^. In addition, we newly show that mitophagy is accompanied by mechanisms of mitochondrial remodeling, both fission–fusion and biogenesis, which sustain increased mitochondrial mass and potential, and boost mitochondrial bioenergetics.

## Results

### Upregulation of autophagy during NGF-induced differentiation

**Fig. 1:**
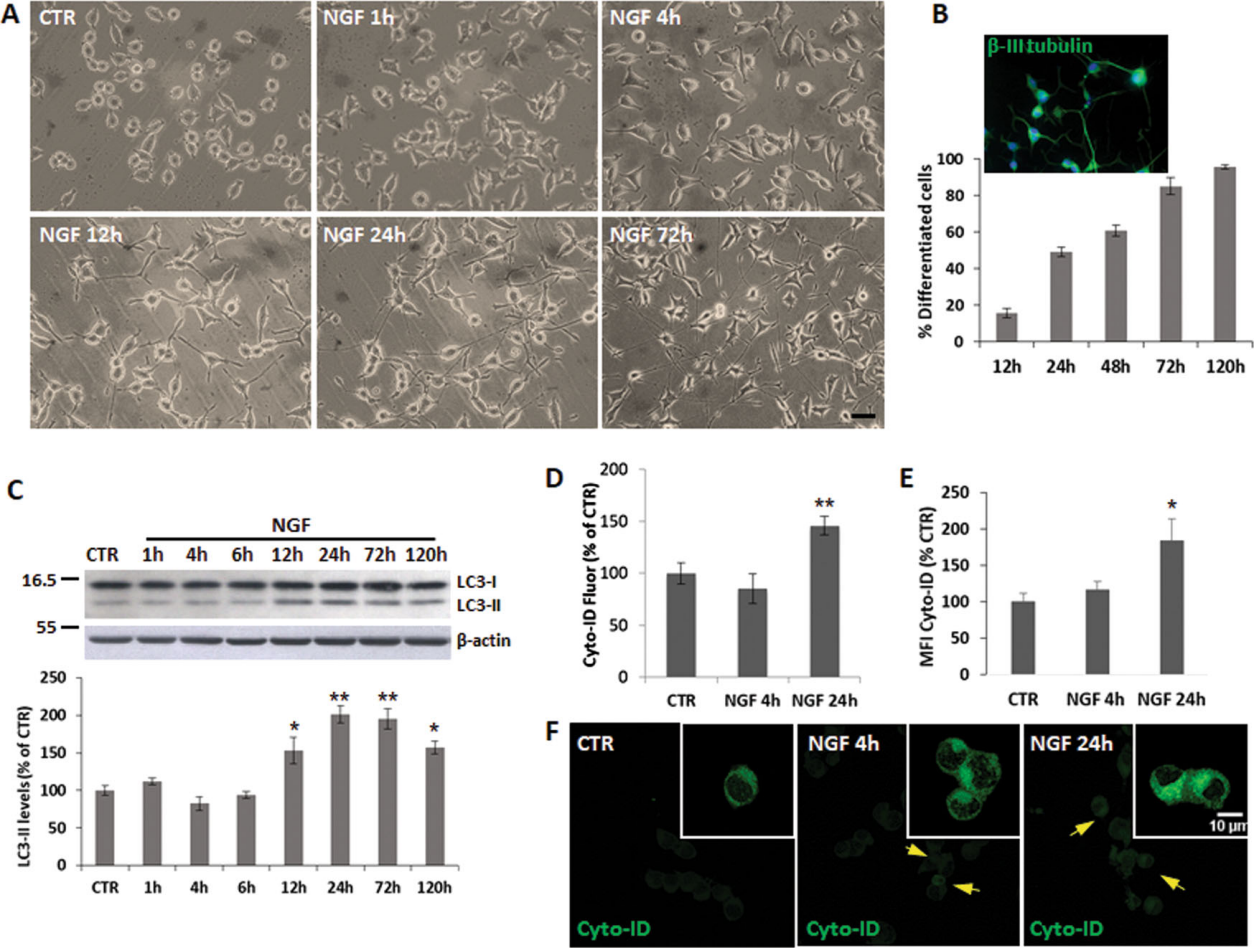
NGF-differentiated PC12 cells show increased autophagy. **a** Representative images of PC12-615 exposed to NGF (10 ng/ml) for 1–4–12–24–72 h. Scale bar = 25 µm. **b** Neuronal PC12 differentiation is measured as percent of cells with neurite processes whose length is at least twice the diameter of cell body. A representative image of β-III tubulin staining is shown in the inset. Data are the mean ± SEM of at least three separate experiments, each performed in duplicate. **c** Densitometric analysis of LC3-II normalized by the β-actin content in PC12-615 cells exposed to NGF (10 ng/ml) for the indicated times. A representative immunoblot of LC3 is shown above. Data, expressed as percent of CTR, are the mean ± SEM of three independent experiments with duplicate samples. **d** Analysis of autophagy by fluorimetry using Cyto-ID® Autophagy detection kit in PC12-615 treated with NGF for 4 or 24 h. Data are the mean ± SEM of two separate experiments, each with three independent samples. **e** Analysis of Cyto-ID® autophagy by fluorescence microscopy in PC12-615 treated with NGF for 4 or 24 h. Data, expressed as percent of CTR, are the mean ± SEM of the mean fluorescence intensities (MFI) normalized by the total number of cells (about 100 cells) in ten random fields from three independent experiments in duplicate. **f** Representative images of Cyto-ID® fluorescence in PC12-615 treated with NGF for 4 or 24 h. Scale bar = 10 µm. ∗*p* ≤ 0.05, ∗∗*p* ≤ 0.01 vs. CTR (ANOVA and Dunnett’s multiple comparisons test)

To investigate mechanisms involved in NGF-induced differentiation, we employed PC12-615 cells overexpressing TrkA receptors^32^, which differentiate more rapidly and in response to lower NGF concentrations^32^ (Fig. 1a, b), compared to PC12wt (Supplementary Fig. S1A–C)^32,33^. To evaluate whether NGF differentiation induces autophagy, we measured LC3-II content^34^. Time-course studies showed that LC3-II levels do not change at short time-points, but start to increase in PC12-615 exposed to NGF (10 ng/ml) for 12 h (Fig. 1c) and remain twofold higher than CTR for 24–120 h, thus showing a trend that correlates with their initial neurite extension and the post-mitotic state (Fig. 1c and Supplementary Fig. S1D, G). These data were confirmed by using the Cyto-ID® Autophagy detection kit. Both fluorimetric analysis (Fig. 1d) and fluorescence microscopy (Fig. 1e, f) show enhanced fluorescence at 24 h, but not at 4 h. NGF-mediated increase of LC3-II is similar to Rapamycin (Rap) and is partially prevented by 3-methyladenine (3-MA) and by wortmannin (WT) (Supplementary Fig. S1E, F, H, I).

**Fig. 2:**
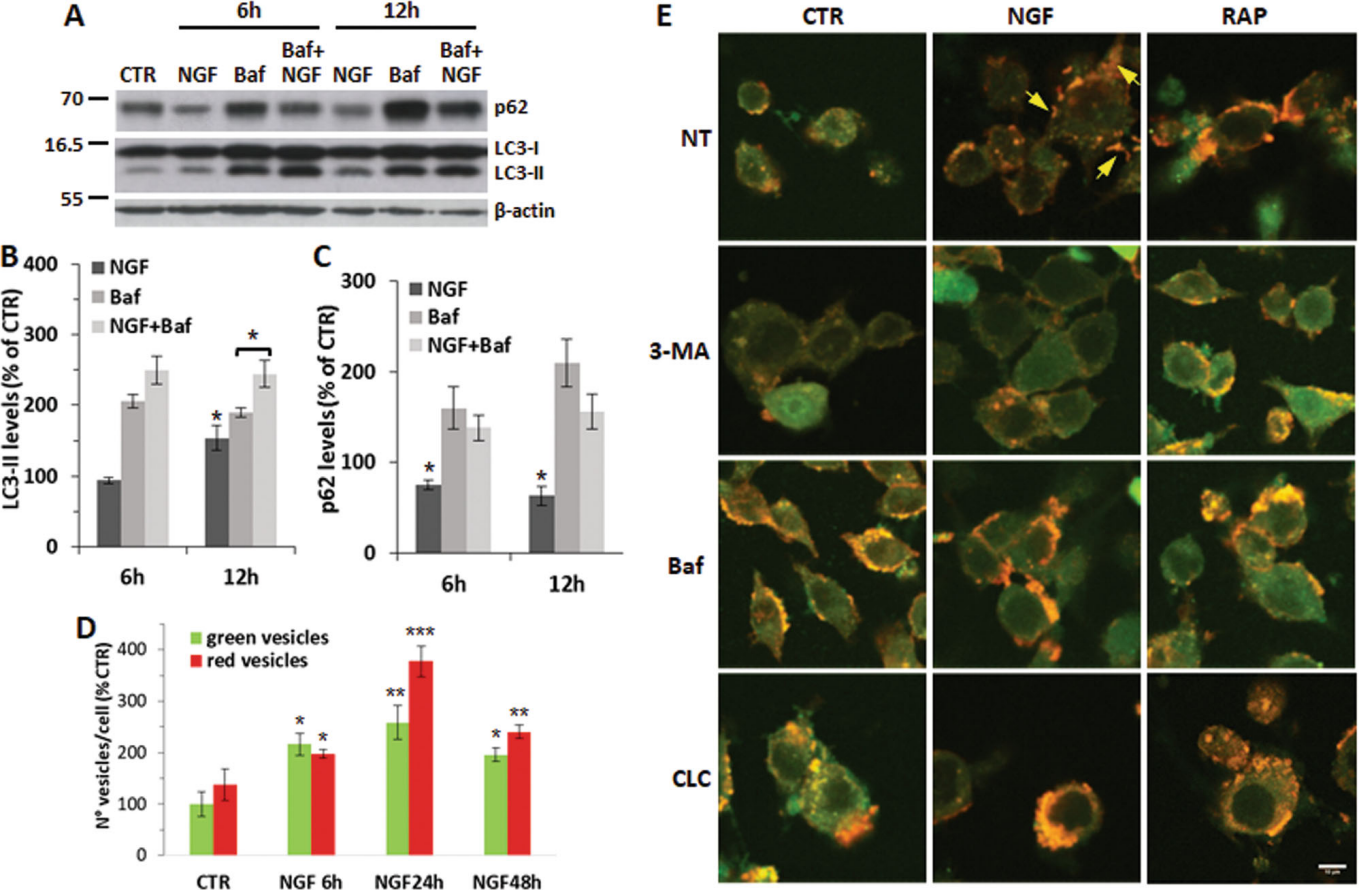
Analysis of the autophagic flux in NGF-treated PC12 cells. **a** Representative immunoblots for LC3-II and p62 content in PC12-615 cells treated for 6 or 12 h with NGF (10 ng/ml) alone or in combination with Baf (100 nM). Values are normalized by the β-actin content. **b–c** Densitometric analysis of LC3-II (**b**) and p62 (**c**) normalized by β-actin. Data are the mean ± SEM of three independent experiments in duplicate. ∗*p* ≤ 0.05 vs. CTR or Baf (*t*-test). **d** Quantitation of green and red vesicles in GFP-RFP-LC3-transfected cells by ImageJ software. Data, expressed as percent of CTR, are the mean ± SEM of the number of vesicles/cell (about 100 cells for each condition) in ten random fields from three separate experiments. ∗*p* ≤ 0.05, ∗∗*p* ≤ 0.01, ∗∗∗*p* ≤ 0.001 vs. CTR (*t*-test). **e** Representative images of GFP-RFP-LC3 fluorescence in PC12 cells treated for 24 h with NGF (10 ng/ml) or Rap (200 nM) alone or in combination with 3-MA (10 mM), or Baf (100 nM) or CLC (1 µM). Scale bar = 10 µm

LC3-II increase may represent either enhanced autophagosome synthesis or blockage of autophagosome degradation^34^. To rule out a block of the autophagosome turnover, we measured LC3-II levels in PC12 cells treated with NGF in the presence of Bafilomycin A1 (Baf). Baf-treated cells show a twofold increase of LC3-II that is further enhanced during co-treatment with NGF for 6–12 h (Fig. 2a, b). The same extracts show a significant reduction of p62/SQSTM1, an adaptor protein that facilitates autophagic degradation of poly-ubiquitinated proteins (Fig. 2a, c), thus supporting the role of NGF in fostering the autophagic flux. A similar trend was found at 24–48 h (Supplementary Fig. S2A–C). The apparent discrepancy between lack of LC3-II accumulation and reduced p62 protein levels at 6 h might reflect a higher degradation rate at this time (Supplementary Fig. S2D), as supported by the upregulation of the lysosomal cysteine protease cathepsin S (Ctss) (Supplementary Fig. S3A).

Finally, the role of NGF in modulating autophagy was confirmed by examining the autophagic flux in PC12 cells expressing a GFP-RFP-LC3 tandem fluorescent protein^34^. Untreated PC12 cells display some diffused fluorescence with a few yellow-green dots. Fluorescence intensity is increased following NGF treatment for 6–48 h (Fig. 2d, e), as determined by orange-red fluorescent vesicles mostly located at growth cones (Fig. 2e, arrows). Induction of autophagosome formation by NGF or by Rap is partially prevented by 3-MA, while their maturation is blocked by Baf and by colchicine (CLC), a microtubule-depolymerizing agent that inhibits transport (and fusion) of autophagosomes to lysosomes (Fig. 2e and Supplementary Fig. S2E).

### Inhibition of autophagy suppresses neuronal differentiation in response to NGF

**Fig. 3:**
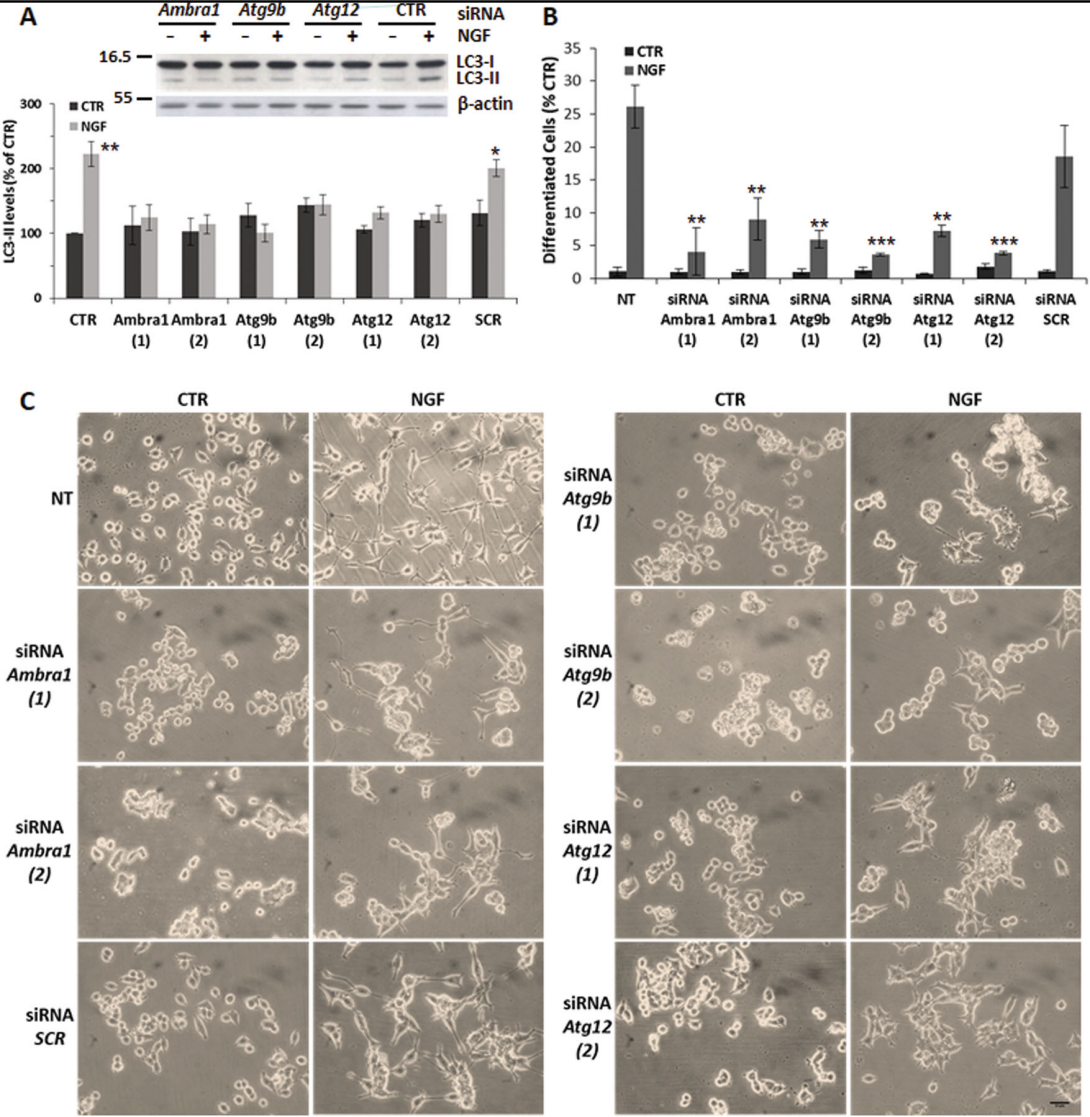
Inhibition of NGF-mediated differentiation by autophagy siRNA knockout. **a** Densitometric analysis of LC3-II/β-actin ratio in PC12-615 cells transfected with siRNA for *Ambra1*, *Atg9b*, or *Atg12*, followed by NGF treatment for 24 h. A representative immunoblot of LC3-II is shown above. Data, expressed as percent of CTR, are the mean ± SEM of three independent experiments with duplicate samples. **b** Quantitation of NGF-induced differentiation in PC12-615 cells transfected with siRNA for *Ambra1*, *Atg9b* or *Atg12* (*SCR* as negative control). Data, expressed as percent of differentiated cells in ten random fields, are the mean ± SEM of three separate experiments. ∗*p* ≤ 0.05, ∗∗*p* ≤ 0.01, ∗∗∗*p* ≤ 0.001 vs. their respective CTR (**a**), or vs. NGF in non-transfected (NT) cells (**b**) (ANOVA and Dunnett’s multiple comparisons test). **c** Representative images of NGF-induced differentiation in PC12-615 cells transfected with siRNA-*Ambra1*, siRNA-*Atg9b*, or siRNA-*Atg12* (si*SCR* as control) followed by exposure to NGF (10 ng/ml) for 24 h. Scale bar = 25 µm

To substantiate the activation of autophagy by NGF, we performed small-interfering RNA (siRNA) knockdown of autophagy-related genes. Reverse transcription PCR (RT-PCR) analysis on NGF-treated cells shows upregulation of Atg9b and Atg12, together with Ambra1, which regulates autophagy during neuronal development^17,23^ (Supplementary Fig. S3A). Their siRNA knockdown decreases protein levels (Supplementary Fig. S3B, D) and causes loss of NGF modulation of LC3-II (Fig. 3a and Supplementary Fig. S4A), as well as of beclin-1 (Supplementary Fig. S4B, D) and p62 (Supplementary Fig. S4C, E). Interestingly, siRNA knockdown studies established the relevance of autophagy in NGF-induced differentiation: both variants of si*Ambra1*, si*Atg9b* and si*Atg12* produce a 52–80% reduction of neurite outgrowth, compared to CTR and siRNA-*SCR* cells (Fig. 3b, c). It is noteworthy that siRNA-transfected cells show a rounded-up morphology and are more prone to cluster. Furthermore, upon NGF treatment most cells display enlarged growth cones, but do not differentiate properly (Fig. 3c). However, siRNA-transfected cells still showed enhanced GAP-43 levels in response to NGF (Supplementary Fig. S4F), suggesting that the block of autophagy does not interfere with NGF regulation of axonal components.

**Fig. 4:**
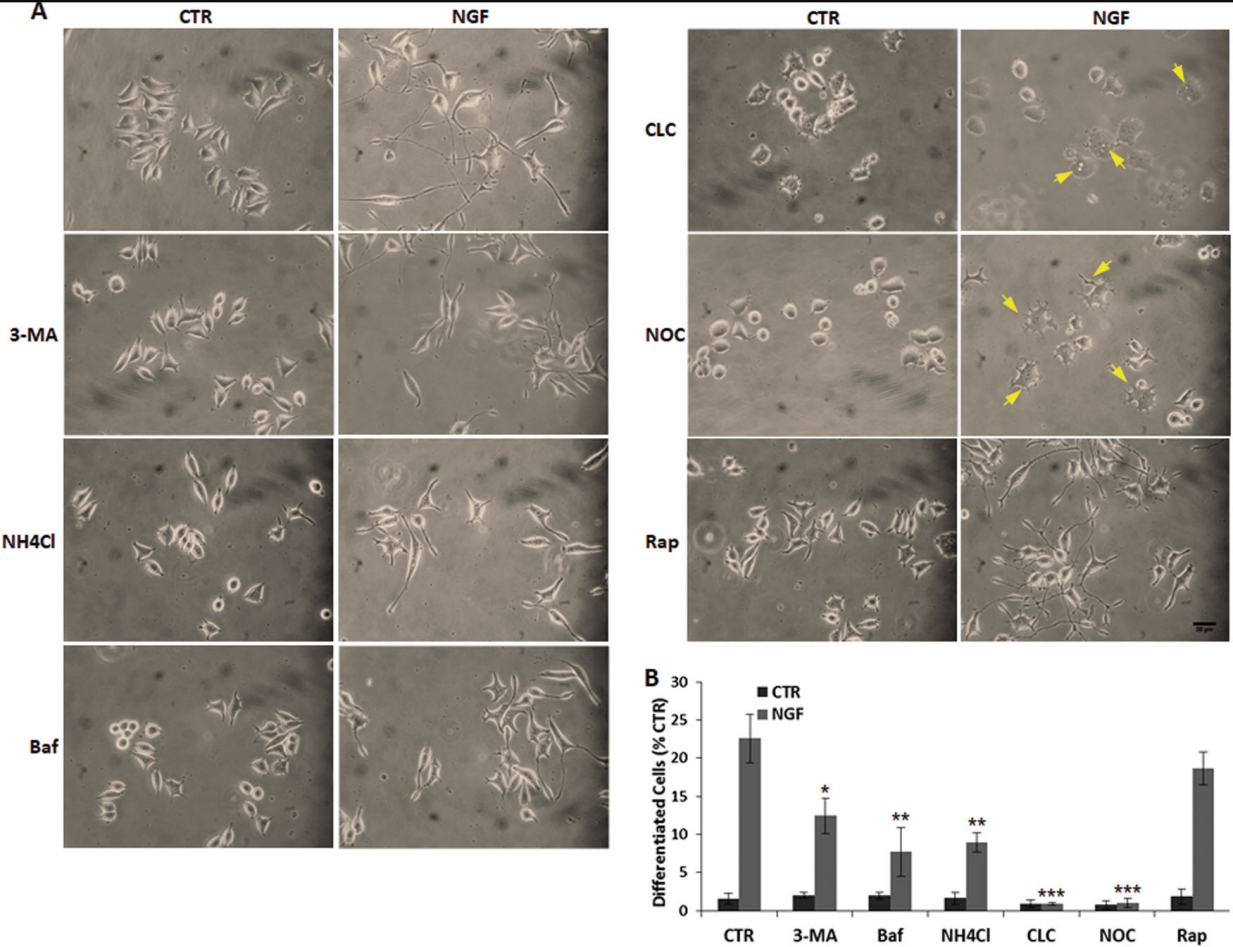
Pharmacological inhibition of autophagy abolishes NGF-mediated differentiation. **a** Representative images of PC12-615 cells following treatment for 24 h with NGF (10 ng/ml) alone or in combination with 3-MA (10 mM), or Baf (100 nM), or NH_4_Cl (12.5 mM), or CLC (1 µM), or NOC (1 µM), or Rap (200 nM). **b** Quantitation of NGF-induced differentiation in PC12-615 cells treated for 24 h with NGF alone or in combination with the autophagy blockers. Data, expressed as percent of differentiated cells in ten random fields, are the mean ± SEM of three separate experiments. ∗*p* ≤ 0.05, ∗∗*p* ≤ 0.01, ∗∗∗*p* ≤ 0.001 vs. NGF (ANOVA and Dunnett’s multiple comparisons test). Scale bar = 25 µm

**Fig. 5:**
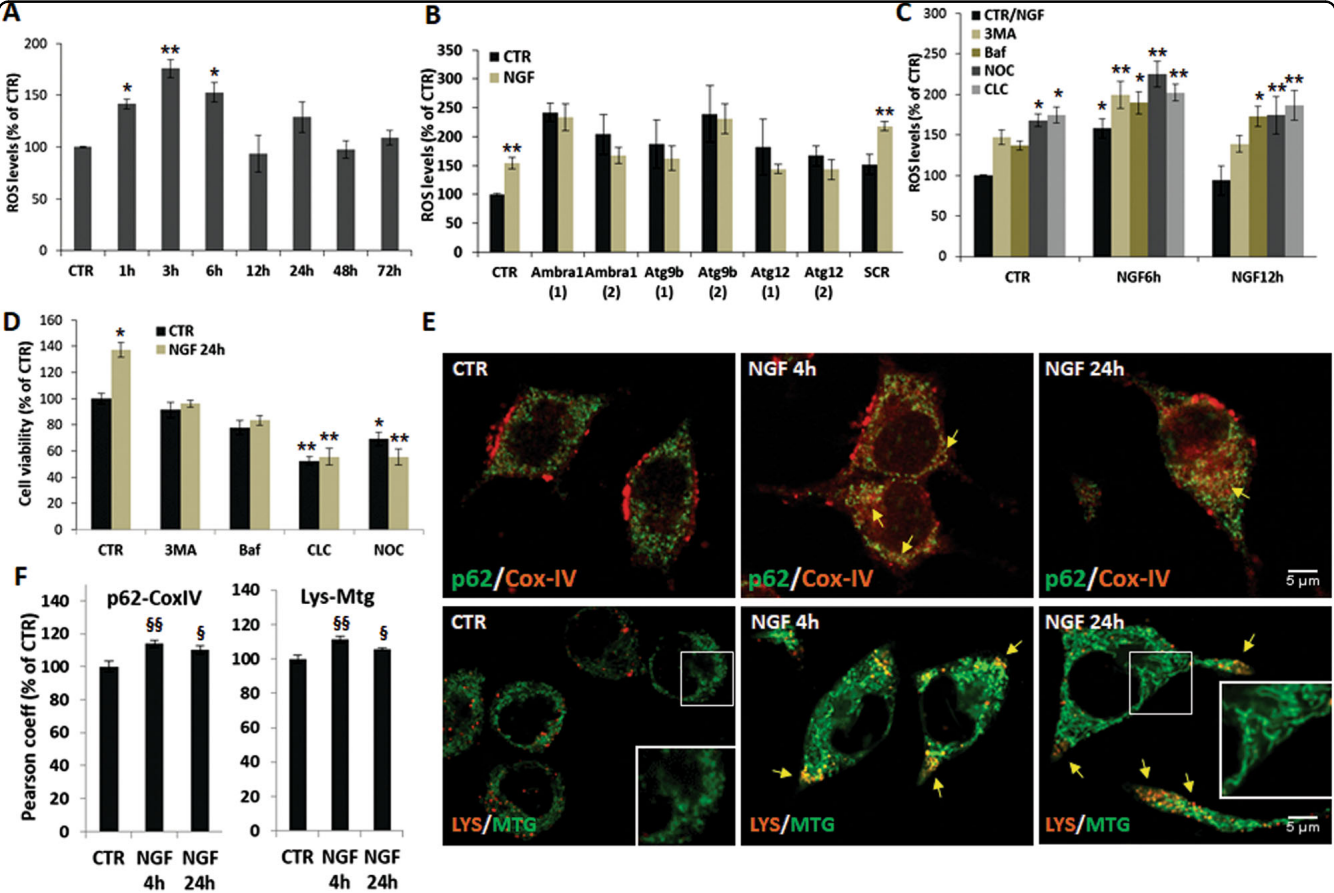
NGF-mediated increase of ROS underlies the induction of mitophagy. **a** Time-course of ROS levels in PC12-615 cells treated with NGF (10 ng/ml) for the indicated times. **b** Effect of *Ambra-1*, *Atg9b*, or *Atg12* siRNA on ROS production after exposure to NGF for 6 h. **c** Effect of autophagy inhibitors on ROS content following NGF treatment for 6 or 12 h. Data in **a**, **b**, **c**, expressed as percent of CTR, are the mean ± SEM of three independent experiments with duplicate samples. **d** Effect of autophagy inhibitors on cell viability after NGF treatment for 24 h. Data, expressed as percent of CTR, are the mean ± SEM of two separate experiments, each with five samples. **e** Representative images of PC12-615 cells treated with NGF (10 ng/ml) for 4–24 h, followed by immunostaining for p62 (green)/CoxIV (red) or addition of LYS and MTG during the last 30 min of incubation. Scale bar = 5 µm. **f** Co-localization of p62/CoxIV or LYS/MTG measured by Pearson coefficient of correlation in about 100 cells for each condition in ten random fields. Data are the mean ± SEM of three independent experiments in duplicate. *p ≤ 0.05, **p ≤ 0.01 vs. their respective CTR (ANOVA and Dunnett’s multiple comparisons test); ^§^*p* ≤ 0.05, ^§§^*p* ≤ 0.01 vs. CTR (*t*-test)

The impact of autophagy on NGF-mediated differentiation was confirmed by its pharmacological inhibition. Autophagy inhibition by 3-MA, or blockage of lysosomal activity (Baf or NH4Cl), or microtubule disruption (CLC or NOC) do not change the morphology of growing cells, but dramatically reduce their ability to differentiate in response to NGF (Fig. 4a, b). It is remarkable that NOC does not alter growth cone formation, but completely blocks neurite extension in response to NGF, while CLC causes extensive vacuolation (Fig. 4a, arrowheads), eventually leading to cell death (Fig. 5d). On the other hand, Rap (200 nM) does not affect NGF response (Fig. 4a, b), suggesting that autophagy is required for NGF-mediated differentiation, which occurs only after the appropriate NGF signaling.

### Calcium and AMPK energy signaling underlie NGF-induced autophagy during differentiation

**Fig. 6:**
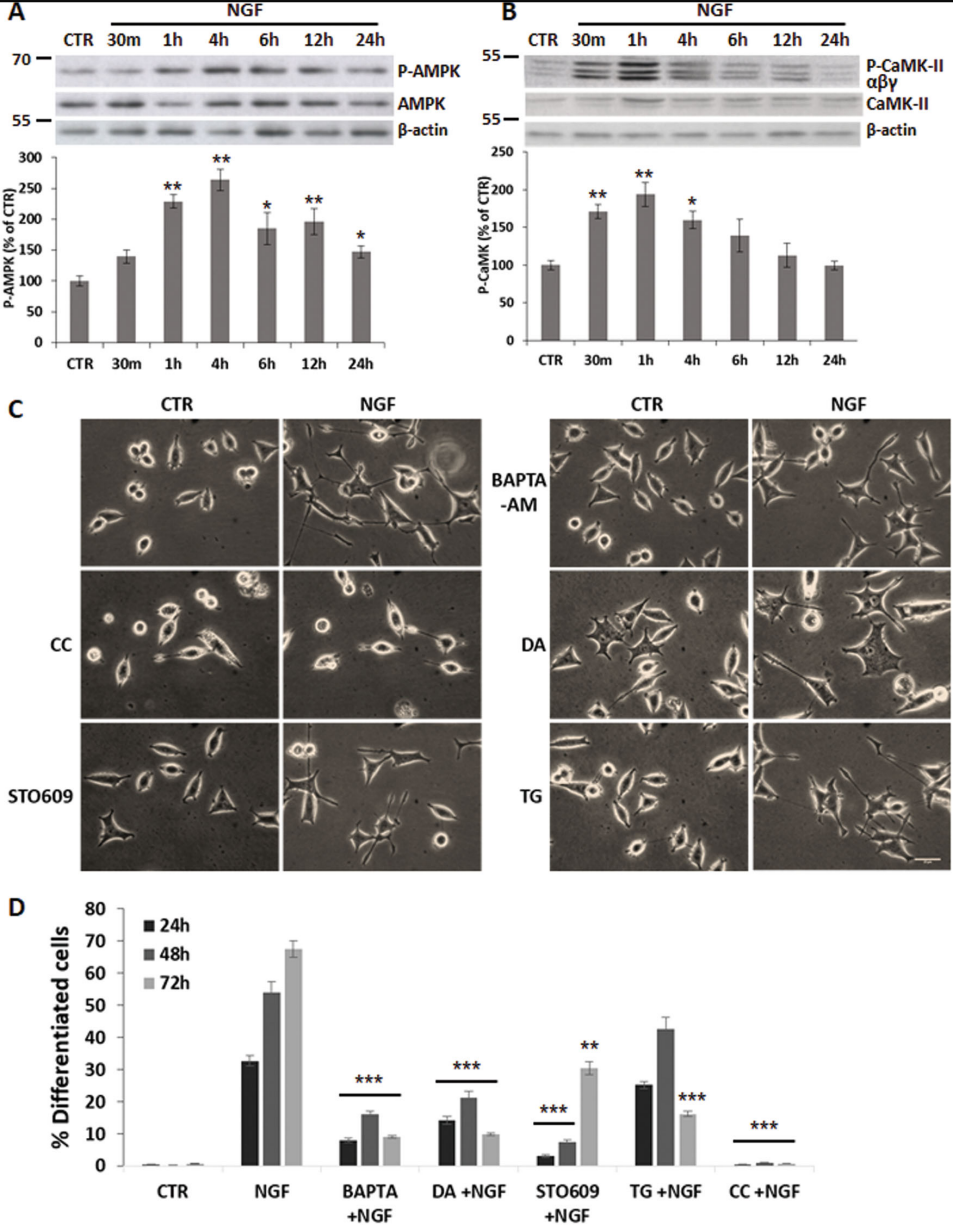
AMPK and CaMK signaling in NGF-mediated differentiation. **a–b** Densitometric analysis and representative immunoblots of P(Thr172)-AMPK (**a**) and P(Thr286)-CaMK(α,β,γ) (**b**) during NGF treatment for the indicated times. Blots were probed for total AMPK and CaMK, respectively, as well for β-actin to normalize for protein content. Data, expressed as percent of CTR, are the mean ± SEM of three independent experiments in duplicate. **c** Representative images of PC12-615 treated for 24 h with NGF (10 ng/ml) alone or in combination with CC (10 µM), STO609 (25 µM), Bapta-AM (1 µM), DA (20 µM), or TG (100 nM). Scale bar = 25 µm. **d** Quantitation of NGF-induced differentiation in PC12-615 cells treated for 24–48–72 h with NGF alone or in combination with kinases inhibitors or Ca^2+^ blockers. Data, expressed as percent of differentiated cells in ten random fields, are the mean ± SEM of three independent experiments in duplicate. ∗*p* ≤ 0.05, ∗∗*p* ≤ 0.01, ∗∗∗*p* ≤ 0.001 vs. CTR (**a–b**) or vs. NGF (**d**) (ANOVA and Dunnett’s multiple comparisons test)

To investigate signaling pathways triggering autophagy during NGF-induced differentiation, we examined the activation of AMPK and mTOR, two kinases known to regulate autophagy^22^. Time-course studies revealed that NGF treatment for 1 h causes a 2.5–3-fold induction of P(Thr172)-AMPK, which remains higher than CTR up to 24 h (Fig. 6a). P-mTOR, instead, is slightly reduced between 12–24 h (Supplementary Fig. S5A), although an opposite trend of both kinases and p62 is observed with higher NGF concentrations (Supplementary Fig. S5B–E).

P(Thr172)-AMPK can be induced by increased AMP/ATP ratio^22^, as well as by N-CaMK-II in response to increased intracellular Ca^2+^ levels^26,27,35^. We found that CaMK is phosphorylated (P-Thr286) by 30 min of NGF treatment (Fig. 6b), in line with previous studies showing that NGF induces the mobilization of intracellular Ca^2+^ from the endoplasmic reticulum (ER) in a TrkA-dependent manner^6^.

To examine the impact of the Ca^2+^-CaMKII and AMPK signaling on PC12 differentiation, we assessed the effect of their pharmacological inhibition. We found that both the AMPK inhibitor compound C (CC) and the selective Ca^2+^/CaMKII inhibitor (STO609) dramatically prevent NGF-induced differentiation (Fig. 6c, d) and GAP-43 expression (Supplementary Fig. S6A). Both molecules inhibit autophagy^26,27^, thus linking both kinases signaling to NGF-induced autophagy during differentiation. Neurite outgrowth is also dramatically reduced by treating cells with NGF in combination with the cell-permeant Ca^2+^-chelator Bapta-AM, or with the ER-Ca^2+^-release inhibitor dantrolene (DA) or, to a lesser extent, with the ER-Ca^2+^-ATPase inhibitor thapsigargin (TG) (Fig. 6c, d), thus confirming the relevance of ER-Ca^2+^ signaling in NGF-induced differentiation^6^. Lack of NGF differentiation during treatment with the pharmacological inhibitors is not due to cell death. Some apoptotic nuclei (1–12%) are found only in cells treated with the Ca^2+^ inhibitors alone, but not during co-treatment with NGF (Supplementary Fig. S6B, C), in agreement with previous findings of NGF-mediated neuroprotection against calcium ion-induced apoptotic cell death^36,37^.

Interestingly, NGF also causes a slight increase of mitochondrial Ca^2+^ in a TrkA-dependent manner in PC12-615, but not in PC12nnr5 (Supplementary Fig. S7A–C). Mitochondrial Ca^2+^ is known to affect the activity of enzymes regulating ATP synthesis, including glycerol-3-phosphate dehydrogenase on the cytoplasmic side of the inner mitochondrial membrane, and pyruvate dehydrogenase, NAD-dependent isocitrate dehydrogenases and α-ketoglutarate dehydrogenase in the mitochondrial matrix^38^. However, the partial effect of Ca^2+^ and CaMKII inhibitors, as compared to CC, clearly suggests the intervention of mechanisms exclusively related to energy balance.

### Altered bioenergetics during NGF-induced differentiation

**Fig. 7:**
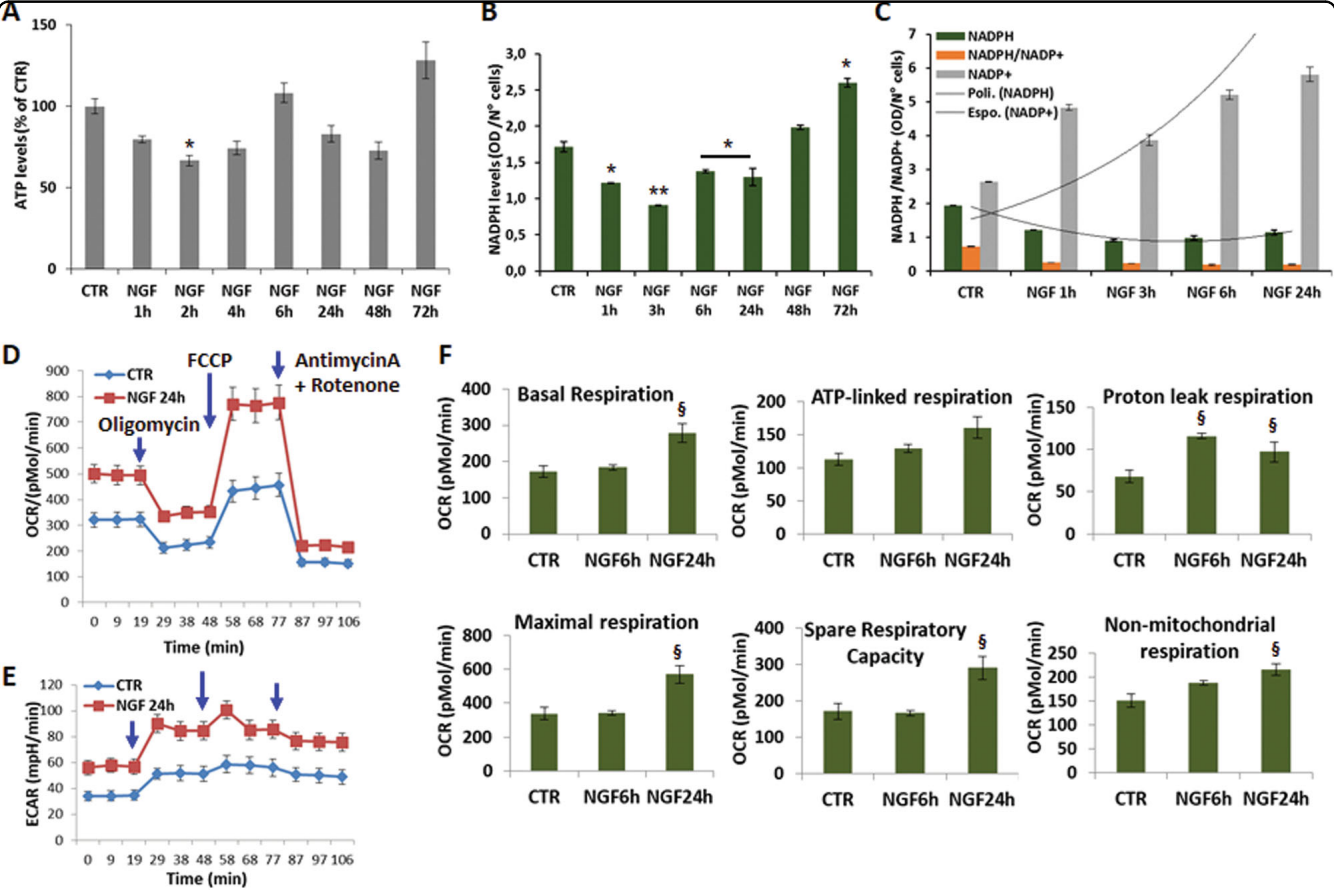
Mitochondrial bioenergetics during NGF-induced differentiation. **a** ATP levels after treatment with NGF (10 ng/ml) for 1–72 h. Data, normalized by the protein content, are the mean ± SEM of three independent experiments in duplicate. **b** NADPH levels during NGF differentiation for 1–72 h. Data, normalized by the protein content, are the mean ± SEM of two separate experiments, each with three independent samples. **c** Modifications of NADPH, NADPH/NADP^+^, and NADP^+^ levels. **d–e** Representative OCR and ECAR profiles, respectively, after exposure to NGF (10 ng/ml) for 24 h. Oligomycin A (1 µM), FCCP (0.8 µM), rotenone (0.5 µM) plus antimycin A (2 µM) were injected at the indicated times to determine the OCR linked to ATP turnover, maximal respiration and proton-leak, respectively. Data are the mean ± SEM of five–six samples, each normalized by the protein content. Similar profiles were obtained in three independent experiments. **f** Changes of bioenergetics parameters after exposure to NGF for 6 or 24 h. Data are the mean ± SEM of three separate experiments, each with five–six independent samples. *p ≤ 0.05, **p ≤ 0.01 vs. CTR (ANOVA and Dunnett’s multiple comparisons test); ^§^*p* ≤ 0.05 vs. CTR (*t*-test)

The increase of P-AMPK is indicative of altered energy homeostasis. Indeed, time-course studies revealed that intracellular ATP levels decrease by 20–35% after 1–2 h of NGF treatment (Fig. 7a) and are fully restored by 72 h, when cells are fully differentiated. To further evaluate the energy status, we measured NADPH/NADP^+^ levels. NADPH, primarily produced in the pentose phosphate pathway, is used in anabolic reactions. We found that NGF causes a dramatic reduction of NADPH (34–50%) and NADPH/NADP^+^ ratio (65%) after 1–3 h (Fig. 7b, c), followed by a net increase at 48–72 h. These data suggest that NGF either decreases ATP and NADPH synthesis, or increases their consumption.

These changes prompted us to assess whether NGF-induced differentiation affects the mitochondrial bioenergetics. Measurement of oxygen consumption rate (OCR) by the extracellular Flux Analyzer showed that NGF treatment for 24 h significantly increases the basal respiration compared to CTR (Fig. 7d–f). NGF-differentiated cells also show higher maximal respiration and spared respiratory capacity (Fig. 7d–f), which are indicative of a greater oxidative capacity, in line with previous findings that neuronal differentiation is associated with mitochondrial biogenesis and a functional reprogramming of mitochondria metabolism^12^. Bioenergetic parameters are not significantly changed at 6 h. At this time, cells display enhanced, although not significant, ATP-linked respiration (Fig. 7f), suggesting that the drop of ATP is not due to decreased mitochondrial efficiency, but rather to its increased utilization. NGF-treated cells also exhibit higher proton-leak and non-mitochondrial respiration that might reflect increased NADPH oxidase activity and ROS production^39^. The higher ATP demand in NGF-differentiated cells was corroborated by a parallel increase of the glycolytic flux, as determined by the extracellular acidification rate (ECAR), in particular after addition of the respiratory chain inhibitors (Fig. 7e). All together, these data suggest that NGF increases mitochondrial function and that the temporary decrease of ATP and NADPH may reflect increased anabolic pathways during differentiation.

### Evidence of mitophagy during NGF-induced differentiation

Several studies suggest that neuronal differentiation involves increased ROS production and signaling, and is prevented by antioxidant molecules^12,40^. FACS analysis of DCFH-DA staining showed that exposure to NGF for 1–6 h causes a 1.5–2-fold increase of ROS, which return to basal levels at later time-points, when cells are differentiated (Fig. 5a and Supplementary Fig. S8A). ROS production in response to NGF is abolished by siRNA knockdown of Ambra1, Atg9b, or Atg12, but not by siRNA-SCR (Fig. 5b and Supplementary Fig. S8B), although basal ROS content in siRNA-transfected cells is higher than CTR. It is remarkable, however, that ROS levels are further enhanced by autophagy inhibitors and blockers, including 3-MA, Baf, CLC, and NOC (Fig. 5c and Supplementary Fig. S8C), as well as NH_4_Cl and PL (data not shown). Nevertheless, NGF-mediated increase of ROS production minimally affects cell viability, which is severely compromised only in the presence of CLC or NOC (Fig. 5d). All together, these data suggest that NGF-induced ROS is not a deleterious event per se, in line with the concept that they might act as signaling molecules during differentiation^12,40^. Moreover, their further increase following genetic or pharmacologic blockade of autophagy suggests that NGF-induced autophagy might be functional to the removal of damaged mitochondria.

This hypothesis has been tested by confocal imaging studies showing a significant increase of p62 localization to mitochondria stained by CoxIV in PC12 cells challenged with NGF for 4–24 h (Fig. 5e, f, arrows), in parallel with a net decrease of p62 fluorescence at 4 h (Supplementary Fig. S9A). These data were supported by the observation that NGF causes a significant increase of Lysotracker-red (LYS) staining at 4–24 h (Supplementary Fig. S9B) and its higher co-localization with Mitotracker-green (MTG), in particular at growth cones tips and along extending neurites (Fig. 5e, f, arrows), confirming that NGF-mediated increase of the autophagic flux (Figs. 1 and 2) involves its functional role in clearing damaged mitochondria.

### NGF-induced differentiation involves modulation of mitochondrial function and dynamics

**Fig. 8:**
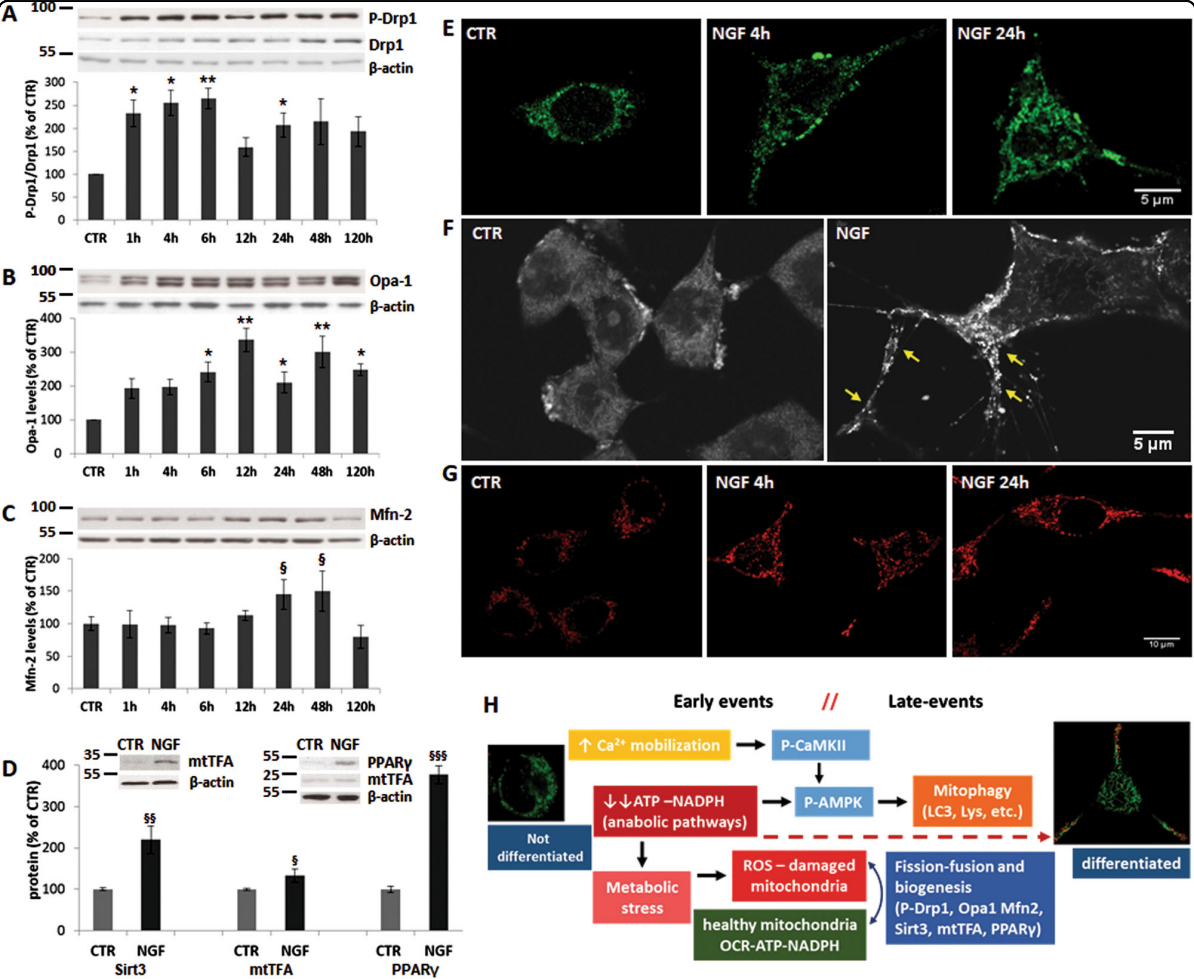
Mitochondrial fission–fusion and biogenesis correlate with higher mitochondrial potential. **a–d** Densitometric analysis and representative immunoblot blot of P(616)-Drp1 and total Drp1 (**a**), Opa1 (**b**), Mfn2 (**c**) after treatment with NGF (10 ng/ml) for the indicated times; **d** levels of Sirt 3, mtTFA, and PPARγ after NGF treatment for 24 h. Representative immunoblots are shown above. Protein levels were normalized by the β-actin content. Data, expressed as percent of CTR, are the mean ± SEM of three independent experiments in duplicate. *p ≤ 0.05, **p ≤ 0.01 vs. CTR (ANOVA and Dunnett’s multiple comparisons test); ^§^*p* ≤ 0.05, ^§§^*p* ≤ 0.01; ^§§§^*p* ≤ 0.001 vs. CTR (*t*-test). **e** Representative images of PC12-615 cells treated with NGF (10 ng/ml) for 4 or 24 h followed by immunostaining for Mfn2. Scale bar = 5 µm. **f** Representative images of mitochondria stained by ethydium bromide after NGF treatment for 24 h. Arrows indicate mitochondria located at growth cones and along neurites. Scale bar = 5 µm. **g** Representative images of MTR staining in PC12-615 treated with NGF for 4 or 24 h. Scale bar = 10 µm. **h** Schematic representation of biochemical and molecular events during NGF-induced differentiation

Alternation of fission/fusion cycles is crucial for mitochondria distribution along axonal branching during differentiation, as well as for their physiological turnover (mitophagy) and adaptation to metabolic perturbations^2,11^. To investigate the role of mitochondrial dynamics during NGF differentiation, we first assessed P-Drp-1, a pivotal protein in mitochondrial fragmentation. We found that NGF treatment stimulates an early induction of P-Drp-1 content (Fig. 8a), in line with a previous finding that NGF-induced axonal branching is reduced when mitochondrial fission is inhibited^2^. Moreover, we newly found that NGF causes a persistent upregulation of the mitochondrial fusion protein Opa1 (Fig. 8b), as well as of Mfn2 content at later times (24–48 h) (Fig. 8c), as confirmed by immunofluorescence staining (Fig. 8e and Supplementary Fig. S9C). It is remarkable that upregulation of fusion proteins is associated with a larger number of mitochondria displaying an elongated tubular morphology (Fig. 5e, insets with enlarged image). These changes are paralleled by enhanced MTG staining in NGF-treated cells (Fig. 5e, and Supplementary Fig. S9B), suggesting that fission/fusion processes are accompanied by a net increase of mitochondrial mass and biogenesis.

To test this hypothesis, we examined molecular events linked to mitochondrial biogenesis and metabolism. Indeed, we found that exposure of PC12 to NGF induces a strong upregulation of Sirt3, a mitochondrial deacetylase known to regulate mitochondrial biogenesis and metabolism (Fig. 8d). Furthermore, NGF causes a dramatic increase of peroxisome proliferator-activated receptor-γ (PPARγ), a transcription factor that regulates glucose and lipid metabolism, as well as a slight but significant induction of the mitochondrial transcription factor mtTFA (Fig. 8d). Levels of Sirt3, PPARγ, and mtTFA also increase at 4 h, as well as the binding activity of PPARγ (data not shown). These data were supported by confocal microscopy studies showing that NGF increases ethydium bromide staining of peripheral mitochondria, in particular those located at growth cones and along neurite branches (Fig. 8f and Supplementary Fig. S9E, F). In addition, we found that NGF treatment boosts the mitochondrial potential, as determined by higher MitoTracker red (MTR) staining (Fig. 8g and Supplementary Fig. S9D), in agreement with the data regarding mitochondrial bioenergetics (Fig. 7). All together, these data provide new evidence that NGF-mediated differentiation involves extensive modulation of mitochondrial dynamics and biogenesis to increase their efficiency and meet higher energy requirements.

## Discussion

Unraveling molecular events involved in neuronal differentiation is crucial for a deeper understanding of brain function. Here, we newly report that NGF-induced differentiation requires the activation of mitophagy through mechanisms that are dependent upon altered energy homeostasis and requirement of mitochondrial remodeling. We show that NGF-dependent neurite outgrowth is strictly correlated with the induction of the autophagic flux (Figs. 1 and 2), as being blocked by its pharmacological inhibition and by siRNA knockdown of autophagy-related genes (Figs. 3 and 4).

In search for mechanistic insights into this process, we considered that neuronal development requires a large amount of energy and building blocks, thereby suggesting that NGF-induced differentiation might influence the bioenergetic status. This working hypothesis was supported by our data of metabolic energy changes, including reduced levels of ATP and NADPH at early stages of NGF differentiation, regardless of increased OCR and related bioenergetic parameters (including ATP-linked respiration) that are indicative of boosted energy metabolism (Fig. 7). Moreover, the enhanced non-mitochondrial respiration may reflect an increase of NADPH oxidase activity and of anabolic reactions to produce amino acids, cholesterol, and fatty acids required for axonal growth. The increase of ECAR during NGF differentiation is also in agreement with previous findings of enhanced glucose metabolism in differentiated PC12 cells and in other neuronal models^12,41^.

The impact of energy metabolism is substantiated by evidence that NGF-induced differentiation causes an early induction of P-AMPK and is fully prevented by CC (Fig. 6). AMPK is activated also in HeLa cells in response to NGF-mediated viability during glucose deprivation^42^. AMPK acts in concert with ULK1 to regulate phosphorylation and localization of ATG9^43^. In line with this finding, NGF-induced differentiation is disrupted in Atg9b-siRNA-transfected cells (Fig. 3). Our data are in accordance with other studies showing the relevance of AMPK signaling in regulating autophagy during neuronal development^18,22,25,44^. AMPK-β1 knockout was found to cause atrophy of dentate gyrus and suppress the differentiation of cultured hippocampal neurons^45,46^.

In addition to metabolic stress, NGF-induced differentiation also correlates with P-CaMKII activation and is significantly prevented by Ca^2+^/CaMKII blockers (Fig. 6), in line with evidence of NGF-dependent Ca^2+^-release from ER^6^. Both AMPK and Ca^2+^/CaMKII inhibition reduce or prevent the induction of the neuronal marker GAP-43 in response to NGF stimulation (Supplementary Fig. S6A). Our data are in agreement with previous reports showing the complex regulation of GAP-43 transcription by Ca^2+^-CaMK, Akt, MAPK-ERK, PKA and PKC, which are activated downstream of NGF signaling^5,47,48^. Both AMPK and Ca^2+^/CaMKII inhibition prevent autophagy^26,27^, supporting the relevance of this process in NGF-induced differentiation. NGF-mediated mobilization of Ca^2+^^6^, as well its buffering by mitochondria (Supplementary Fig. S7) might activate mitochondrial dehydrogenases and enhance oxidative phosphorylation^38^. However, the intense mitochondria activity makes them more prone to produce ROS, which accumulate in non-dividing cells. Hence, the need to foster the turnover of overworked ROS-producing mitochondria by mitophagy, as determined by enhanced p62-CoxIV and LYS-MTG co-localization (Fig. 5)^49^. In addition, ROS levels further increase in siRNA-transfected cells, as well as in cells treated with autophagy inhibitors (Fig. 7).

The transient increase of ROS produced by NGF (Fig. 5) is similar to that observed in other models of neuronal differentiation^12,40,50^. It has been proposed that ROS might act as second messenger, since differentiation is prevented by antioxidant molecules^12,40,51^. It is conceivable that ROS increase following enhanced energy metabolism might serve to induce mitophagy for mitochondria quality control and remodeling in post-mitotic neurons^13,29,52,53^. We can speculate that this function might be achieved through Atg9 downstream of AMPK signaling, since Atg9L2(Atg9b) harbors a putative mitochondrial localization signal, although not experimentally characterized^53,54^.

The increase of mitophagy is accompanied by higher P-Drp-1 levels (Fig. 8), based on the role of mitochondrial fission in the fragmentation of damaged overused mitochondria that must be cleared by mitophagy^53–55^. On the other hand, enhanced fragmentation facilitates translocation of mitochondria along growing neurites, in agreement with its role in axonal branching^2^, and is paralleled by early-upregulation of Opa1 and later induction of Mfn2 (Fig. 8). The increase of mitochondrial fusion, together with upregulation of mitochondrial biogenesis, might reflect the net increase of mitochondrial mass and the appearance of elongated networked mitochondria (Fig. 5), as well as the increase of mitochondrial potential and bioenergetics (Figs. 7 and 8).

Overall, we here provide the first evidence of a functional link between boosted energy metabolism and molecular events modulating mitophagy, and mitochondrial biogenesis and remodeling during NGF-induced differentiation. Our data are in agreement with a previous study showing metabolic reprogramming and mitochondrial biogenesis during maturation of cortical neurons^12^ and might represent an extension of those findings to a specific NGF model. As depicted in Fig. 8h, we can schematically identify two major sets of events at early and late stages that flow into one another during NGF differentiation. From a dynamic perspective, we can speculate that the first set of changes might be caused by Ca^2+^-CaMKII signaling, since Ca^2+^ mobilization occurs in milliseconds (Supplementary Fig. S7) and P-CaMKII is induced after 30 min of NGF treatment. Mitochondrial Ca^2+^ buffering might increase proton-leak and ROS generation, thus activating mitochondrial fragmentation and mitophagy. A second set of events might be more specifically linked to metabolic perturbations due to decreased ATP/NADPH used up in anabolic pathways and converging on P-AMPK to keep energy metabolism, mitophagy, and mitochondrial dynamics active throughout the entire process of differentiation. Enhanced metabolism, together with mitochondrial fission–fusion and biogenesis, ensure a constant remodeling of mitochondria to fit morphological and functional changes of post-mitotic neurons.

In conclusion, we show that NGF-dependent differentiation occurs through a complex signaling network timely and functionally related to the control of energy and redox homeostasis in response to higher energy demand to meet the morphological remodeling of post-mitotic neurons. The functional relevance of these interactions is fascinating, and further studies will be needed to better dissect this process in more details and obtain a metabolomic profile. NGF is essential in neuroprotection in the central and peripheral nervous system, through its anti-gliosis activity^56–58^. Moreover, NGF can regulate different stages of neuronal precursor maturation during neurogenesis in the subventricular zone^59^, which might explain why NGF differentiation involves Ambra1-mediated autophagy^24^. Therefore, uncovering mechanisms underlying NGF-mediated modulation of mitochondrial function might be relevant to both neurogenesis and mechanism of regeneration following brain injury.

## Materials and methods

### Cell cultures and treatments

PC12 cells (clone 615) overexpressing the TrkA receptor^32^ were kindly provided by MV Chao (Skirball Institute, New York University School of Medicine, NY). PC12wt and PC12-615 cells were maintained in Dulbecco’s modified Eagle medium (DMEM) supplemented with 10% fetal bovine serum, 5% heat-inactivated horse serum, 2 mM l-glutamine, 100 µg/ml streptomycin, 100 U/ml penicillin, 200 µg/ml G418, in a humidified atmosphere of 95% air 5% CO_2_ at 37 °C, as previously described^10^. All cell culture reagents were purchased from EuroClone (Milano, Italy). PC12 differentiation was achieved by using murine 2.5 S NGF (mNGF, Promega Inc., Madison WI, USA) purified from male mouse submaxillary glands. Differentiation was measured also following treatments with BAPTA/AM (Invitrogen, ThermoFisher Scientific) or Thapsigargin (Invitrogen, ThermoFisher Scientific). Wortmannin, 3-MA, rapamycin, bafilomycin A1, pepstatin, leupeptin, colchicine, nocodazole, dantrolene, compound C, STO609 were all purchased from Sigma-Aldrich.

### Mitochondrial function and morphology

Mitochondrial mass and potential were assessed by using MitoTracker Red/Green (Molecular Probes Inc., Eugene OR, USA) staining, indicators of mitochondrial potential and mass/morphology, respectively. Briefly, cells (2 × 10^4^/well) were grown onto poly-l-lysine-coated coverslips and exposed to the specific treatments. Cells were loaded with 20 and 200 nM MitoTracker Red and Green, respectively, or 1 µM rhodamine-123, or 1 µM ethydium bromide during the last 30 min of treatment and then rinsed twice with PBS. Coverslips were mounted with Dako Fluorescent Mounting Medium (Dako Agilent Technologies, Santa Clara CA, USA) and analyzed by fluorescence microscopy. Images were captured at 360 magnification (Plan Apo objective; 360 oil) using a motorized Nikon Eclipse 90i (Nikon, Tokyo, Japan) fluorescence microscope equipped with a CCD camera (Hamamatsu-CoolSnap, Hamamatsu Corporation, Tokyo, Japan), or by confocal microscopy using a Nikon Eclipse Ti inverted microscope and Nikon A1 confocal microscope with a 60 × Plan Apo oil immersion objective. NIH ImageJ and NIS-Element AR analysis software were used for image analysis and processing. Images were taken with the same parameters and fluorescence intensity of cells was measured and then averaged to obtain the mean fluorescence intensity (MFI). On average, about 100–150 cells for each condition were analyzed in about ten randomly picked fields. Data, expressed as percent of CTR, are the mean ± SEM of three separate experiments, each performed in duplicate.

### Immunofluorescent staining

PC12 cells were grown onto 12 mm poly-l-lysine-coated coverslips (2 × 10^4^ cells/well). After treatments, cells were washed with PBS, fixed with 4% paraformaldehyde, permeabilized with 0.5% Triton X100 in PBS and incubated with the blocking solution (10% normal goat serum) followed by overnight incubation with the following primary antibodies: rabbit SQSTM1/p62 (1:100, Cell Signaling Technologies), mouse CoxIV (1:200, Cell Signaling Technologies), rabbit Mfn2 (1:100, Cell Signaling Technologies), or mouse β-III tubulin (1:1000, Abcam, Cambridge, UK). After washing with PBS, coverslips were incubated for 2 h at room temperature with the goat anti-rabbit Alexa 488 or goat anti-mouse Alexa 546 conjugated antibodies (1:500; Molecular Probes, Invitrogen, Carlsbad, CA). After washes, nuclei were counterstained for 1 min with DAPI (100ng/ml) or Hoechst 33342 10 µg/ml for 15 min. Coverslips were mounted with Dako Fluorescent Mounting Medium (Dako Agilent Technologies) and analyzed by fluorescence microscopy, as described above.

### Autophagy detection

Autophagic activity was detected by using the Cyto-ID™ Autophagy Detection Kit (ENZO Life Sciences). Briefly, PC12-615 cells (2.5 × 10^5^/well) were trypsinized, pelleted by centrifugation and washed in assay buffer. Cells were resuspended in 250 µl of freshly diluted Cyto-ID reagent and incubated at 37 °C for 30 min, followed by two washes and resuspension in 500 µl of assay buffer. The Cyto-ID fluorescence was immediately measured at excitation/emission wavelengths of 480 nm/530 nm, respectively, using a Cary Eclipse Fluorescence Spectrophotometer (Agilent Technologies). The autophagic flux was measured by Lysotracker-red staining (Molecular Probes) by adding Lysotracker-red (1 µM) during the last 30 min of treatment. Coverslips were mounted with Dako Fluorescent Mounting Medium (Dako Agilent Technologies) and analyzed by fluorescence microscopy, as described above.

### Plasmid DNA and transfection

Plasmid DNA encoding mRFP-GFP tandem fluorescence-tagged LC3 (ptf-LC3 plasmid, ID 21074)^60^ was obtained from Addgene (Cambridge, MA). The GFP-RFP-LC3 protein allows for discrimination between autophagosomes and autolysosome based on GFP sensitivity to the acidic pH of lysosomes. To monitor for LC3 cleavage by lysosome proteases, PC12 cells (2 × 10^4^ cells/well) were plated onto 12 mm poly-l-lysine-coated coverslips and treated with the transfection mix containing 500 ng of plasmid DNA and Lipofectamine 2000 (Life Technologies) for 6 h. After transfection, cells were washed three times with culture medium and grown for 24 h before exposure to the specific treatments. After treatments, cells were washed with PBS and coverslips were mounted with Dako Fluorescent Mounting Medium (Dako Agilent Technologies) and analyzed for the number of vesicles/cell by fluorescence microscopy.

Transfection of siRNAs was performed using Metafectene Pro (Biontex, Martinsried, Germany) using the siRNAs sequences for Ambra1, Atg9b, and Atg12 reported in Supplementary materials. A scrambled sequence was used as a control.

### Bioenergetics by Seahorse technology

Mitochondrial OCR was determined by using a Seahorse XF24 Extracellular Flux Analyzer (Seahorse Bioscience, Copenhagen, Denmark). PC12-615 cells were seeded in XF plates 48 h prior to the assay. Cells were treated with NGF for 6–24 h and then analyzed by using the Seahorse XF Cell Mito Stress Test Kit (Seahorse Bioscience) according to manufacturer instructions. Three measurements of OCR and ECAR were taken for the baseline and after sequential injection of mitochondrial inhibitors. The ATP synthase inhibitor Oligomycin A (1 mM), the ATP synthesis uncoupler carbonyl cyanide-4-trifluoromethoxyphenylhydrazone FCCP (0.8 µM), the complex I inhibitor rotenone (0.5 µM) and complex III inhibitor antimycin A (2 µM) were used to determine OCR parameters. OCR and ECAR from each well were normalized by the protein content by using the Bradford assay (Bio-Rad, Hemel Hempstead, UK).

### NADP/NADPH assay

NADP and NADPH levels were detected using NADP/NADPH Quantitation Colorimetric Kit (BioVision Inc., Milpitas CA, USA) according to the manufacturer's protocol. Briefly, cells (1.5 × 10^6^/well) were lysed by sonication (two cycles: 5 s for five pulses at 70% power) in 800 µl of extraction buffer. Samples (125 µl) were then directly used to detect total NADP/NADPH. To detect NADPH only, samples were heated at 60 °C for 30 min to clear out all NADP^+^ species. Colorimetric measurements were performed at OD 450 nm using a Cary 60 ultraviolet–visible spectrophotometer (Agilent Technologies).

### ATP determination

PC12-615 cells were plated into 6-well plates (7 × 10^4^ cells) and treated with NGF (10 ng/ml) for 1–72 h. Cells were subsequently lysed by using lysis buffer and ATP activity was analyzed by using the adenosine 5′-triphosphate (ATP) Bioluminescent Assay Kit (Sigma-Aldrich) according to manufacturer instructions. The light intensity was measured with a luminometer (Lumat LB9507, Berthold) in a 5 s time period and expressed as relative light units/μg of protein.

### ROS analysis by flow cytometry

Determination of intracellular levels of total ROS was carried out by flow cytometry using 2′,7′- dichlorodihydrofluorescein-diacetate (H_2_DCFDA, Molecular Probes), as previously described^61^. Cells (2 × 10^5^ cells/well) were plated in 6-well plates (EuroClone) precoated with poly-l-lysine (0.1 mg/ml). DCFH-DA was added during the last 30 min of treatments. Cells were harvested with 0.08% Trypsin and analyzed by FACS (FACScan Becton-Dickinson, San Jose, CA) using the Cell Quest Software (BD Bioscience). Fluorescence was measured on 1 × 10^4^ cells and data were analyzed by using the Flowing Software 2.5.1 (Turku Center for Biotechnology, University of Turku, Finland).

### Western blot analysis

Total cell extracts and western blotting were performed as previously described^10,61^. Cell lysates were prepared in lysis buffer containing proteases inhibitors (Mini EDTA-free Protease Inhibitor Cocktail, Roche Applied Science, Sussex, UK) and phosphatases inhibitor cocktail (PhosSTOP, Roche Applied Science). Protein concentration was determined by using the Bradford assay (Bio-Rad, Hemel Hempstead, UK).

Total proteins (25 μg) were separated on 10–12% sodium dodecyl sulfate polyacrylamide gel electrophoresis gels and transferred to nitrocellulose Protran™ (PerkinElmer, Waltham MA, USA). After blocking, blots were probed overnight at 4 °C with the primary antibody in Tris-buffered saline 0.1% Tween-20 (TBST), followed by incubation for 1 h at room temperature with HRP-conjugated donkey anti-rabbit or anti-mouse IgG (1:5000; GE Healthcare Life Sciences, Buckinghamshire, UK) for 1 h at room temperature. The following antibodies were used for western blots: rabbit phospho(Thr172)-AMPKα (1:1000), total AMPKα (1:1000), rabbit phospho(Thr286)-CaMKII (1:1000), total CaMKII (1:1000), rabbit phospho(Ser2448)-mTOR (1:1000), total mTOR (1:1000), rabbit LC3B (1:1000), rabbit Beclin-1 (1:1000), rabbit SQSTM1/p62 (1:1000), Phospho(Ser616)-Drp1 (1:1000), total Drp1 (1:1000), rabbit mitofusin-2 (1:100), rabbit Sirt3 (1:1000), mouse β-actin (1:1000), rabbit phospho(Ser473)-Akt (1:1000), total Akt (1:1000) were all purchased from Cell Signaling Technologies (Beverly MA, USA). Mouse Opa1 (1:1000) was from BD Biosciences (Franklin Lakes NJ, USA). Rabbit mtTFA (1:1000), rabbit PPARγ (1:1000), rabbit Ambra1 (1:1000), rabbit Atg9b (1:1000), and rabbit Atg12 (1:1000) were from Santa Cruz Biotechnology, Dallas TX, USA). Mouse anti-GAP-43 (1:1000) was from Sigma. HRP-conjugated donkey anti-rabbit (1:5000) and anti-mouse IgGs (1:5000) were purchased from GE Healthcare Life Sciences (Little Chalfont, Buckinghamshire, UK). All immunoblots were probed for β-actin, to normalize for protein content. Detection was carried out by using the enhanced chemiluminescence system (ECL, GE Healthcare Life Sciences). Quantification of bands was performed by densitometry using NIH ImageJ software.

### Statistical analysis

All data are presented as the mean ± SEM of the number of independent samples in separate experiments, as indicated in the figure legends. Statistical analysis was performed by using GraphPad Prism 6.0 (GraphPad Software, La Jolla, CA, USA). All quantitative data were analyzed by one-way ANOVA and Dunnett’s multiple comparisons test for multiple treatments or by Student’s *t*-test for single comparisons (*p ≤ 0.05, **p ≤ 0.01, ***p ≤ 0.001 vs. CTR), as indicated in the figure legends. For morphology analyses, individual images of CTR and treated cells were assembled and the same adjustments were made for brightness, contrast, and sharpness using Adobe Photoshop (Adobe Systems, San Jose, CA).

## Electronic supplementary material

Revised Supplementary material(DOCX 3227 kb)
