# Supplementary material for: Differentiation by nerve growth factor (NGF) involves mechanisms of crosstalk between energy homeostasis and mitochondrial remodeling
Source: Cell Death Dis. 2018 Mar 9;9(3):391. doi: 10.1038/s41419-018-0429-9 (PMC5844953; doi:10.1038/s41419-018-0429-9)
Supplement: Supplementary file 1 — Revised Supplementary material(DOCX 3227 kb) [file 41419_2018_429_MOESM1_ESM.docx]

**Differentiation by Nerve Growth Factor (NGF) involves mechanisms of crosstalk between energy homeostasis and mitochondrial remodeling** by F Martorana, D Gaglio*, MR Bianco*, F Aprea, A Virtuoso, M Bonanomi, L Alberghina, M Papa, AM Colangelo

**Supplementary Figure S-1**


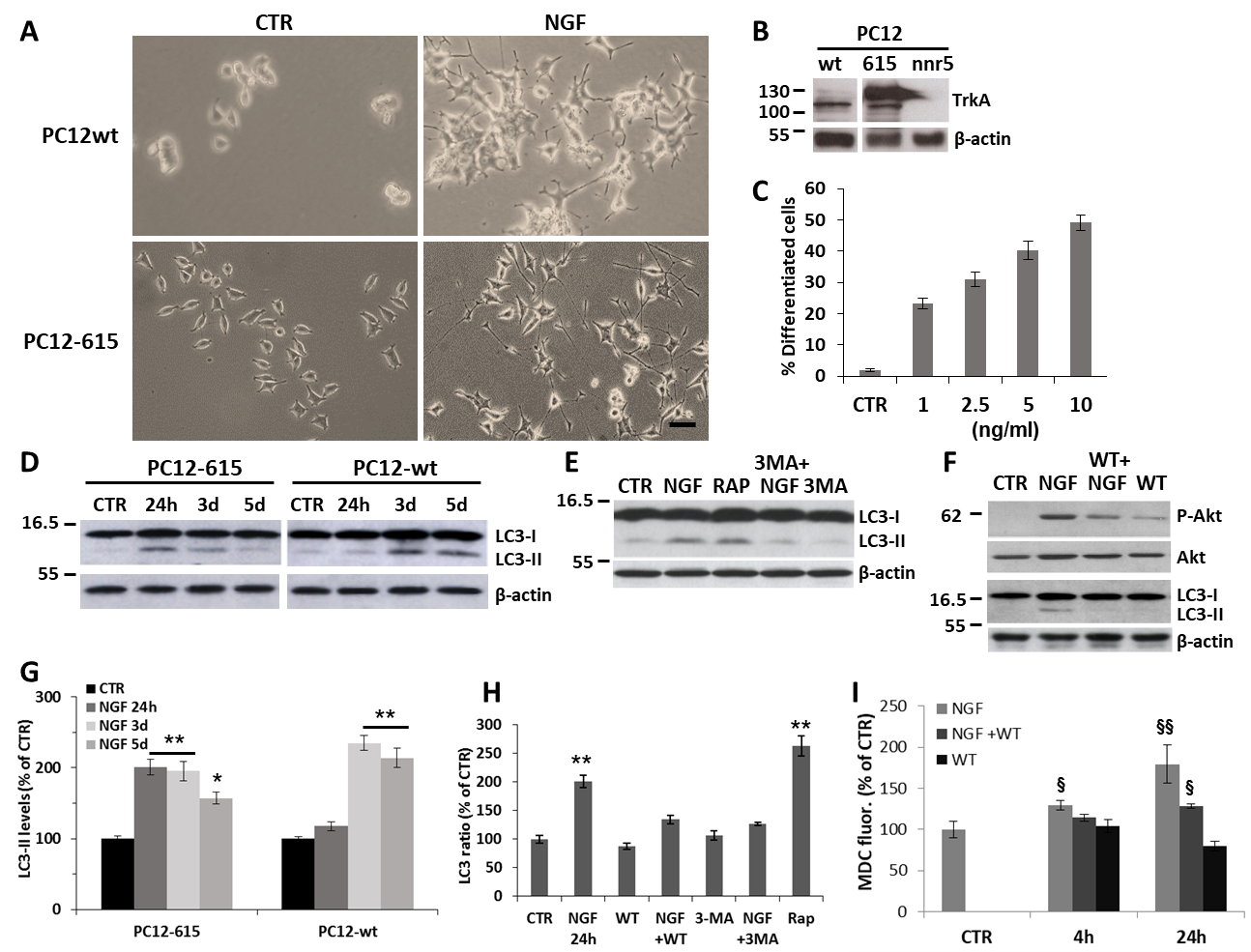


**Figure S1. Comparison of autophagy in PC12wt and PC12-615. A**) Representative images of PC12-wt and PC12-615 cells exposed to NGF (50 or 10ng/ml, respectively) for 72h. Scale bar = 25 µm. **B**) Expression levels of TrkA in PC12 wt, 615 and nnr5. **C**) Dose-response of PC12-615 differentiation with NGF (1, 2.5, 5 or 10 ng/ml) for 24h. Data are the mean ±SEM of three experiments with duplicate samples. **D**) Representative western blot for comparative analysis of LC3-II content in PC12-615 and PC12wt cells treated with NGF (10 or 50 ng/ml, respectively) for 24h or 3-5 days. LC3-II levels are comparable, but delayed in PC12wt. **E**) Representative immunoblots of LC3-II in PC12-615 treated for 24h with NGF (10ng/ml), alone or in combination with 3-methyladenine (3-MA, 10 mM). The effect of NGF is similar to that induced by Rapamycin (Rap, 200 nM). **F**) Representative immunoblots of LC3-II and P-Akt and total Akt in PC12-615 treated with NGF (10ng/ml), alone or in the presence of wortmannin (WT, 200 nM). **G-H**) Densitometric analysis of LC3-II normalized by the β-actin content in PC12-615 and PC12wt cells treated with NGF (10 or 50 ng/ml, respectively) for 24h or 3-5 days (**G**), and the effect of 3-MA and WT inhibition (**H**). Data in G-H are the mean ±SEM of three independent experiments in duplicate. **I**) Fluorimetric analysis of monodansylcadaverine (MDC) staining in PC12-615 treated with NGF (10 ng/ml) for 4 or 24h. A partial reduction of acidic vacuoles is found when cells are preincubated for 10 min with WT. ∗ 𝑝≤ 0.05, ∗∗ 𝑝≤ 0.01 versus CTR (ANOVA and Dunnett’s multiple comparisons test). § 𝑝≤ 0.05, §§ 𝑝≤ 0.01 versus CTR (*t*-test).

**Supplementary Figure S-2**


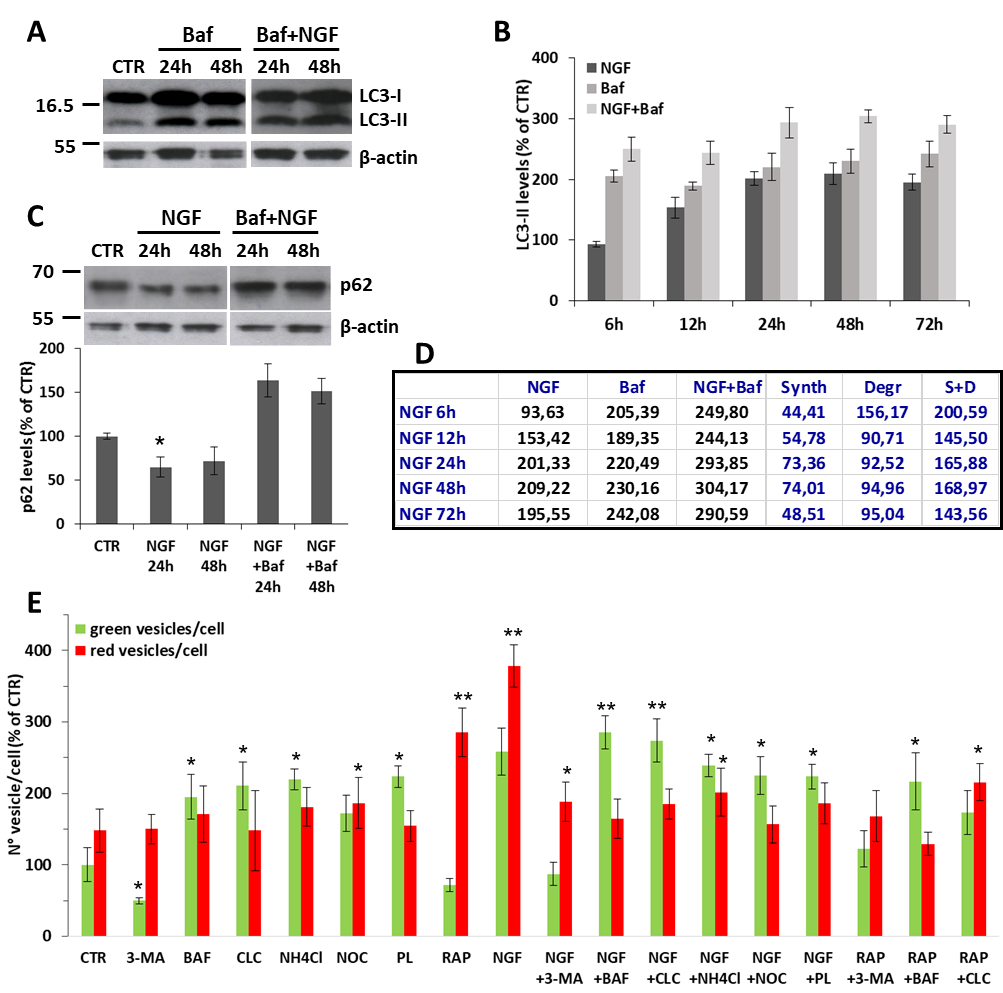


**Figure S2. Analysis of the autophagic flux in NGF-treated PC12 cells.** **A**) Representative western blot of LC3-II levels in PC12-615 cells treated for 24-48h with NGF (10 ng/ml) alone or in the presence of Baf (100 nM). **B**) Quantitation of LC3-II/β-actin ratio during the entire time-course 6-72h. Data, expressed as percent of CTR, are the mean ±SEM of three independent experiments in duplicate. **C**) Representative immunoblot and quantitation of p62 in PC12 cells treated for 24-48h with NGF (10 ng/ml) alone or in the presence of Baf (100 nM). Data are the mean ±SEM of two independent experiments in duplicate. **D**) The box shows the mean data and the corresponding rates of LC3-II synthesis and degradation at 6-12-24-48-72h of NGF treatment. **E**) Quantitation of green and red vesicles in GFP-RFP-LC3- transfected PC12 cells treated with NGF (10 ng/ml) or Rap (200 nM) alone or in combination with 3-MA (10mM), or Baf (100 nM), or CLC (1 µM) (Figure 2E), or Nocodazole (NOC, 1µM), or NH4Cl (12.5mM) or a mix of the lysosomal inhibitors pepstatin/leupeptin (PL, 10 µM). Data, expressed as percent of CTR, are the mean ±SEM of vesicles normalized by the total number of cells (about 100 cells for each condition) in 10 randomly picked fields from three independent experiments. ∗ 𝑝≤ 0.05, ∗∗ 𝑝≤ 0.01 versus CTR (*t*-test).

**Supplementary Figure S-3**


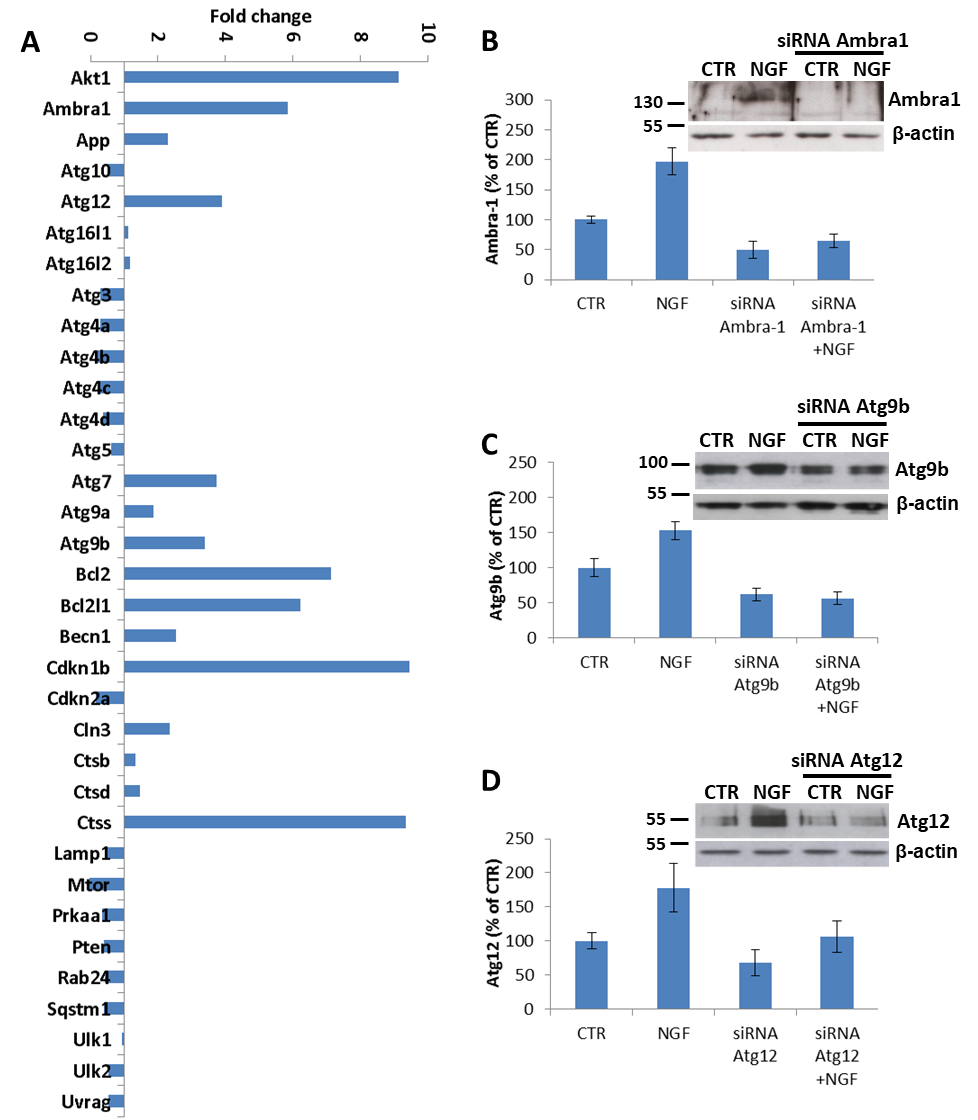


**Figure S3. Expression of autophagy genes in response to NGF. A**) RT-PCR array for autophagy genes on PC12-615 treated with NGF for 6h. Data, expressed as fold of induction (ΔΔCt), are the mean of two independent experiments. In addition to induction of Akt1 and Bcl-2, NGF increases Ambra1, as well as of Atg7, Atg9b and Atg12, which are involved in autophagosome biogenesis and elongation. Moreover, NGF increases Cyclin-dependent kinase inhibitor 1B (Cdkn1b, p27Kip1), a cell cycle inhibitor during NGF differentiation (Bianco et al., 2011), and cathepsin S (Ctss), a lysosomal cysteine protease. **B-D**) Expression levels and representative immunoblot of Ambra-1, Atg9b and Atg12 after NGF treatment for 24h. Blots were probed for β-actin to normalize for protein content. siRNA knockdown, which reduces protein content by about 30-50% (si-Ambra1, 50.3%; si-Atg9b, 38.4%; si-Atg12, 31.6%), abolishes the NGF-mediated induction of protein content.

**Supplementary Figure S-4**


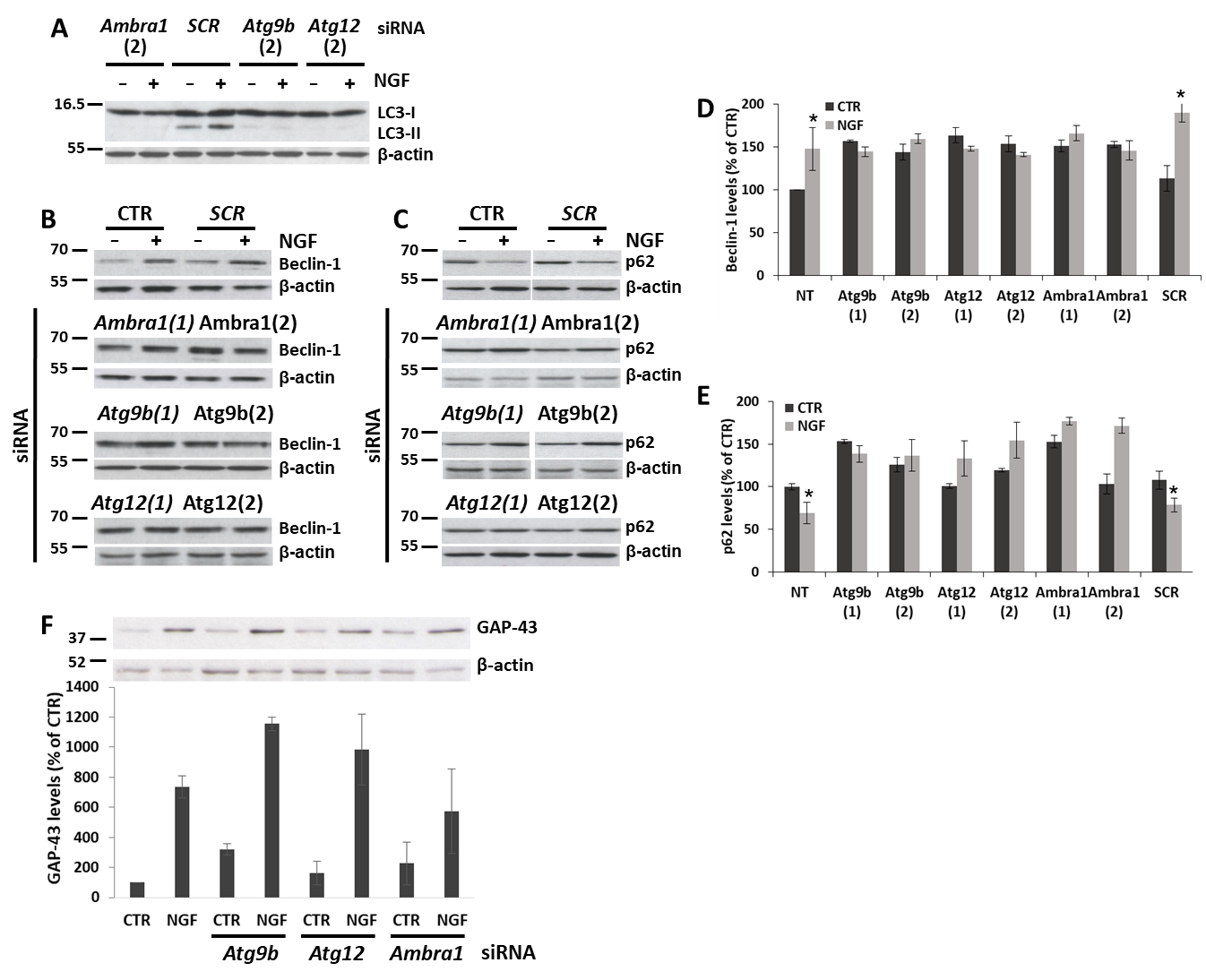


**Figure S4. Inhibition of NGF-mediated autophagy by siRNA knockout of autophagy genes**. **A**) Representative western blot of LC3-II in PC12-615 cells transfected with siRNA-*Ambra1*(2), siRNA-*Atg9b*(2), or siRNA-*Atg12*(2), followed by NGF (10 ng/ml) treatment for 24h. **B-C**) Representative immunoblots of beclin-1 (B) and p62 (C) in PC12-615 cells transfected with siRNA-*Ambra1* (1-2), or siRNA-*Atg9b* (1-2), or siRNA-*Atg12* (1-2) or SCR, followed by NGF treatment for 24h, as compared to non-transfected (CTR) cells. Blots were probed for β-actin to normalize for protein content. All siRNA were run on parallel gels. **D-E**) Densitometric analysis of beclin-1 (D) and p62 (E) in PC12-615 not-transfected (NT) or siRNA transfected cells followed by NGF treatment for 24h. NGF fails to regulate LC3-II (A), beclin-1 (B and D) and p62 (C and E) in siRNA transfected cells, but not in NT or scrambled siRNA (siSCR) used to control for non-specific effects of siRNA transfection. Data, expressed as percent of CTR, are the mean ±SEM of three independent samples. ∗𝑝 ≤ 0.05 versus CTR (*t*-test). **F**) Expression levels and representative immunoblot of GAP-43 in PC12-615 treated with NGF for 24h. NGF-mediated induction of GAP-43 levels is not changed by siRNA knockdown of *Ambra-1*, or *Atg9b* or *Atg12*, as compared to not transfected cells. A slight increase of GAP-43 is seen in the CTR of transfected cells, most likely due to the transfection procedure, as observed in siRNA-SCR transfected cells (data not shown). Data are the mean of four independent samples.

**Supplementary Figure S-5**


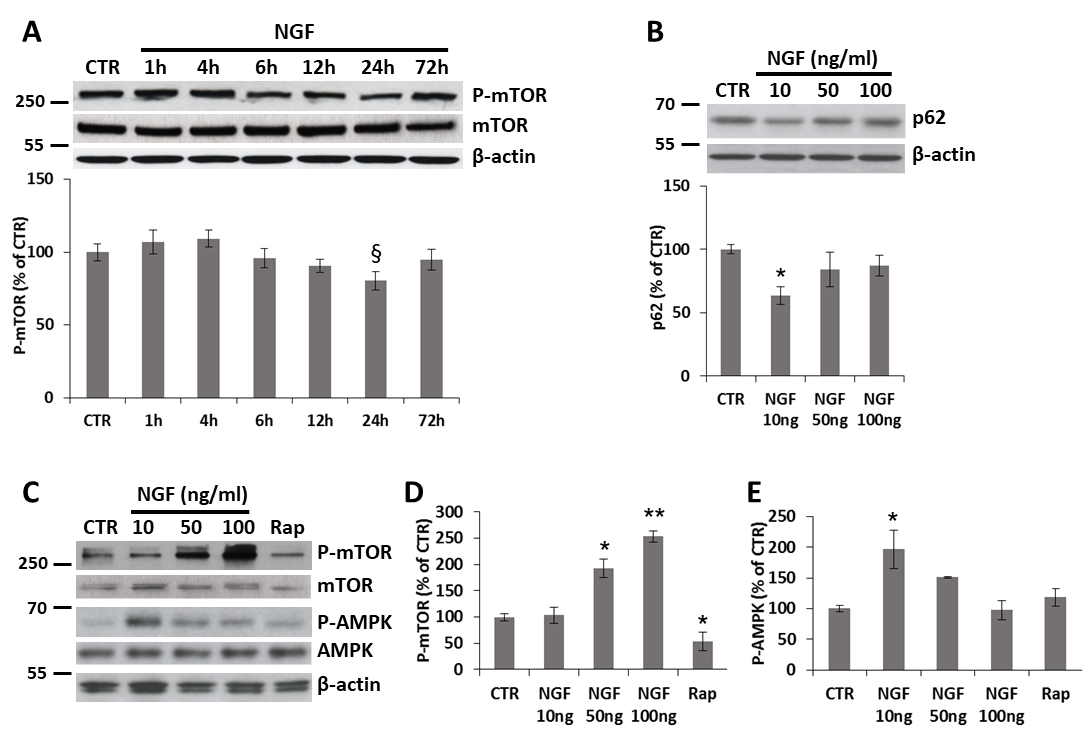


**Figure S5. The effect of NGF on P-mTOR and P-AMPK is dose-dependent. A**) Densitometric analysis and representative immunoblot blot of P(Ser2448)-mTOR and total mTOR after treatment with NGF (10 ng/ml) for the indicated times. Data are the mean ±SEM of three experiments in duplicate. § p≤ 0.05 versus CTR (t-test). **B**) Dose-response and representative immunoblot for p62 content in PC12-615 cells treated with NGF 10-50-100 ng/ml for 4h. High NGF concentrations (50 or 100 ng/ml) do not change p62 levels. **C**) Representative blots of P-mTOR, total mTOR, P-AMPK and total AMPK in PC12-615 cells treated with NGF 10-50-100 ng/ml for 4h. Blots were probed for β-actin to normalize for protein content. High concentrations of NGF (50 or 100 ng/ml) cause a dose-dependent increase in P-mTOR, inversely correlated with the decrease of P-AMPK levels. Rap (200 nM) is used as a control. **D-E**) Dose-response of P-mTOR (D) and P-AMPK (E) in PC12-615 cells treated with NGF 10-50-100 ng/ml for 4h. Data in B and D-E represent the mean ±SEM of three independent samples. * p≤ 0.05, ** p≤ 0.01 versus CTR (ANOVA and Dunnett’s multiple comparisons test). § 𝑝 ≤ 0.05 versus CTR (*t*-test).

**Supplementary Figure S-6**


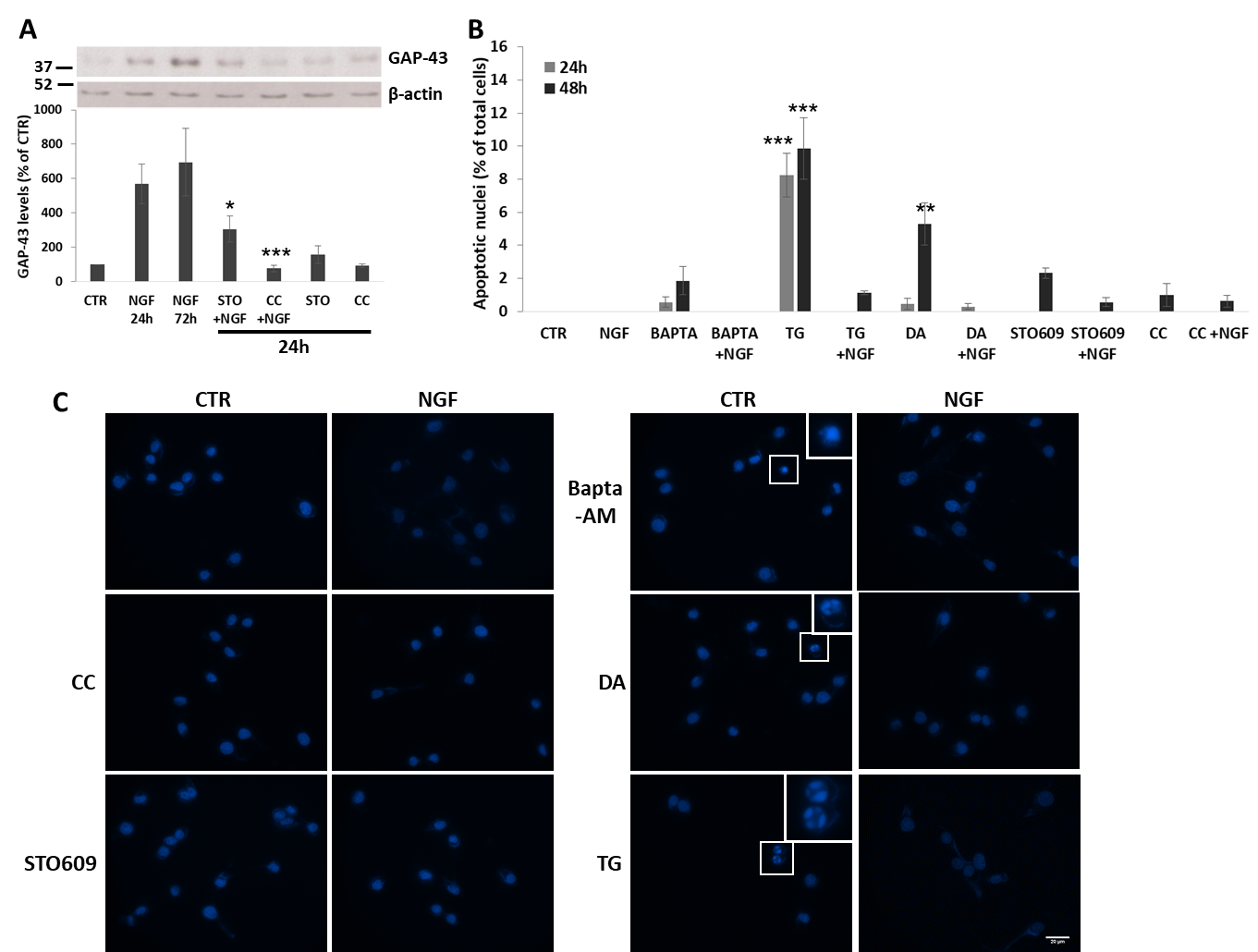


**Figure S6. Effect of kinases inhibitors and Ca^2+^ blockers on cell survival during NGF-induced differentiation. A**) Expression levels and representative immunoblot of GAP-43 in PC12-615 treated with NGF for 24-72h. Blots were probed for β-actin to normalize for protein content. NGF-mediated induction of GAP-43 is partially decreased by Ca^2+^/CaMKII inhibitor STO609 (25 µM) and fully prevented by CC (10 µM), as compared to NGF at 24h. Data are the mean of three separate experiments with duplicate samples. **B**) Quantitation of apoptotic nuclei during NGF differentiation (24-48h) in the presence of kinases inhibitors or Ca^2+^ blockers. Data, expressed as percent of total cells in 10 random fields for each sample, are the mean ±SEM of three independent experiments with duplicate samples. **C**) Representative images of Hoechst-33342 staining of apoptotic nuclei in PC12-615 cells treated for 24h with NGF (10ng/ml) alone or in combination with CC (10 µM), STO609 (25 µM) Bapta-AM (1 µM), DA (20 µM) or TG (100 nM). Scale bar = 20 µm. ∗ 𝑝≤ 0.05, ∗∗ 𝑝≤ 0.01, ∗∗∗ 𝑝≤ 0.001 versus CTR (ANOVA and Dunnett’s multiple comparisons test).

**Supplementary Figure S-7**


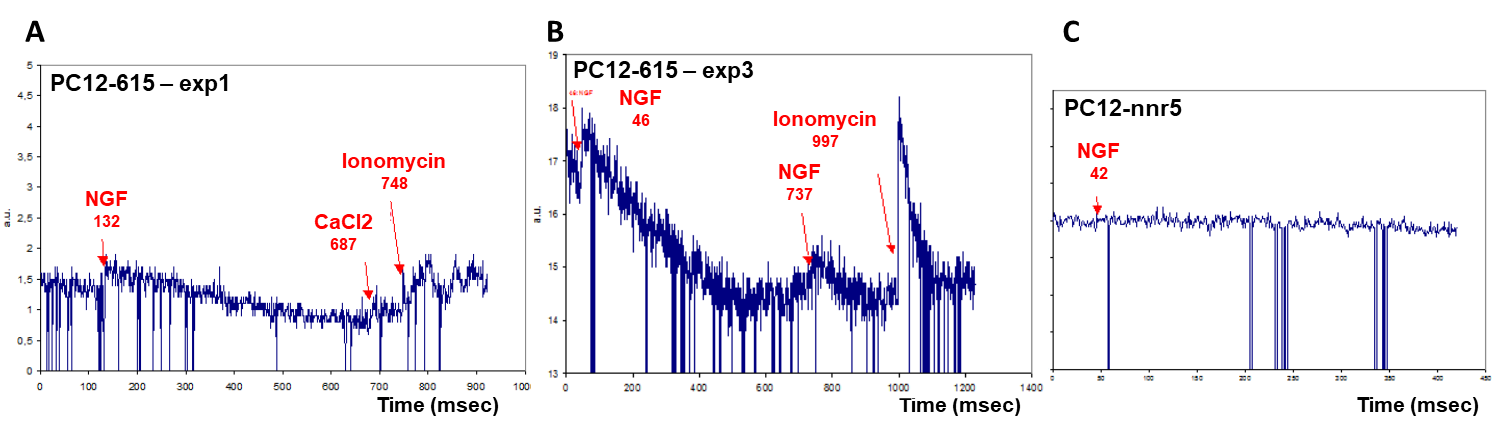


**Figure S7. NGF promotes mitochondrial Calcium entry. A-C**) Representative profiles of mitochondrial Ca^2+^ entry after NGF treatment in PC12-615 (**A-B**), as compared to PC12 nnr5 (**C**) used as negative control (PC12nnr5 do not express TrkA, but only the p75 receptor). Arrows indicate the time of addition of NGF at 132 msec, after baseline stabilization (**A**), or 46 msec and 737 msec (**B**), or 42 msec (**C**), as well as times of addition of CaCl2 (687 msec in A) or ionomycin (748 msec in A, 997 msec in B).

**Supplementary Figure S-8**


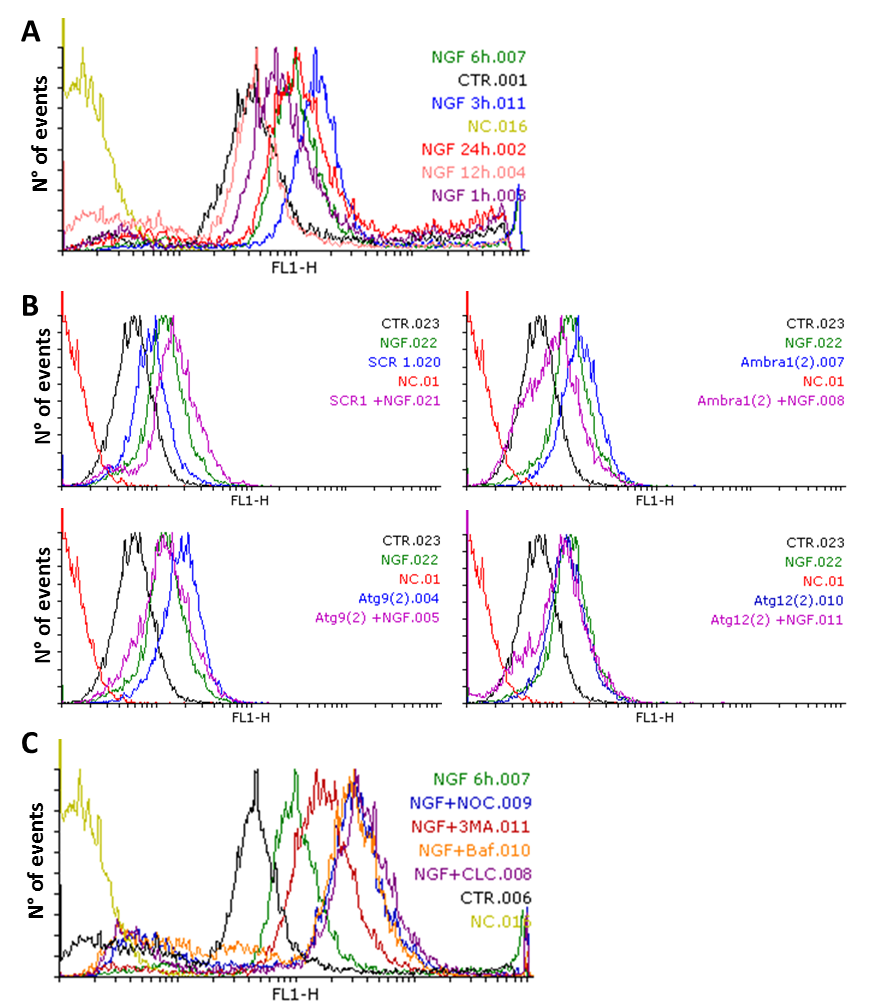


**Figure S8. ROS levels in PC12 treated with NGF. A-B-C**) Representative FACS profiles of DCFH-DA staining in: **A**) PC12 treated with NGF (10 ng/ml) for the indicated times; **B**) PC12 transfected with *Ambra-1*, *Atg9b* or *Atg12* siRNA and treated with NGF (10 ng/ml) for 6h; **C**) PC12 treated with NGF (10 ng/ml) alone or in combination with the indicated autophagy inhibitors for 6h.

**Supplementary Figure S-9**


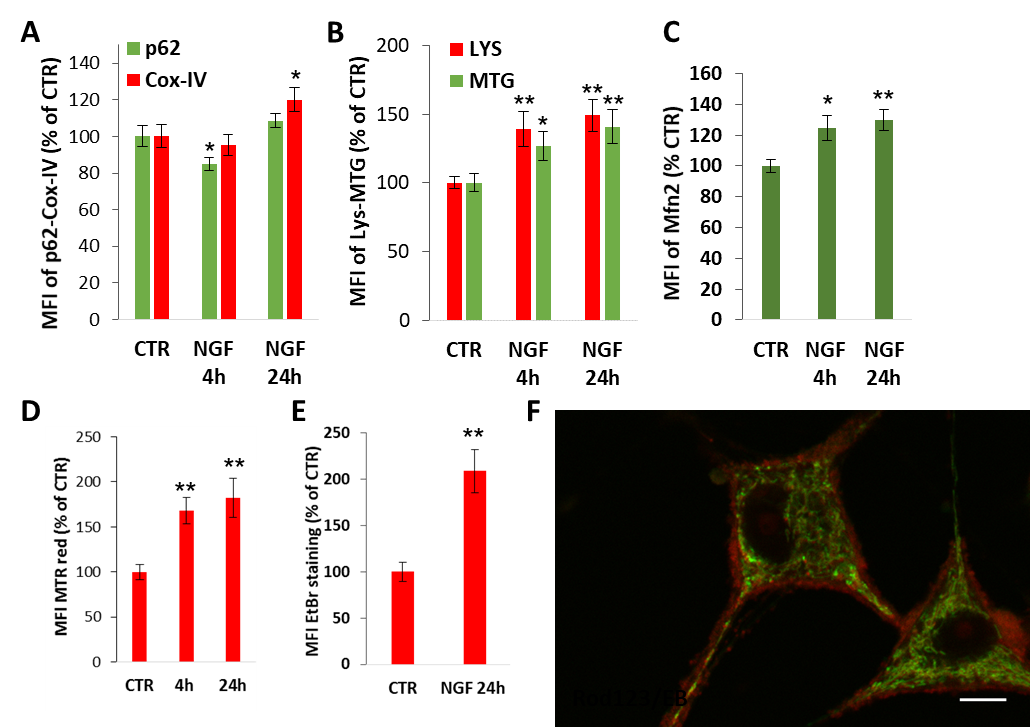


**Figure S9. Quantitation of mitophagy and mitochondrial dynamics. A-B-C**) Quantitation of p62/CoxIV (**A**), LYS/MTG (**B**) and Mfn2 (**C**) fluorescence expressed as mean fluorescence intensity (MFI) normalized by the total number of cells. **D**) Mitotracker red (MTR) fluorescence measured as MFI normalized by the total number of cells. On average, about 100 cells for each condition were counted in about 10 randomly picked fields for all fluorescence imaging studies. Data, expressed as percent of CTR, are the mean ±SEM of three experiments with duplicate samples. * p≤ 0.05, ** p≤ 0.01 versus CTR (*t*-test). **E**) Quantitation of Ethydium bromide fluorescence expressed as MFI/cell. **F**) Representative image of mitochondria morphology, by differential labeling with Rhodamine 123 (a cationic, green fluorescent dye that is sequestered by active mitochondria) and Ethydium Bromide (red). In NGF-treated cells most of central mitochondria appear tubular, while peripheral mitochondria are mostly fragmented and labeled by EB. Scale bar = 5 µm.

**Supplementary Materials and Methods**

**Cell viability**

Cell survival was assessed by MTT assay (Sigma). The reduction of the yellow tetrazolium salt (MTT) to the purple formazan is dependent on the activity of mitochondrial dehydrogenases by intact mitochondria and can also be taken as an index of mitochondrial activity. Briefly, PC12-615 cells (8000 cells/well) were plated in 96-well plates (Euroclone) pre-coated with poly-L-lysine (0.1 mg/ml). Following treatments, tetrazolium salts (0.5 mg/ml) were added directly to the culture medium for 2h at 37°C in a humidified atmosphere. After incubation, dimethyl sulfoxide (DMSO) was added into the wells, and plates were agitated for 15 minutes. The absorbance of samples was measured at wavelength 570 nm (630 nm background wavelength) with a Microplate Reader (Bio-Rad, Hercules, CA). MTT conversion levels were expressed as a percentage of control.

**Hoechst 33342 staining assay**

Apoptotic cell death was detected by monitoring morphological changes of nuclei using Hoechst 33342 staining. Briefly, PC12 cells were grown onto 12mm poly-L-lysine-coated coverslips (2x10^4^ cells/well). After treatments, cells were washed with PBS three times and stained with Hoechst 33342 (10 µg/ml) for 10 min at room temperature in the dark. Coverslips were mounted with Dako Fluorescent Mounting Medium (Dako Agilent Technologies). Images were acquired at 60X magnification using a fluorescence microscope (Nikon Eclipse 90i) equipped with a CCD camera. NIH ImageJ software was used for image analysis and processing. Apoptotic cells were counted in 10 random fields for each sample, and expressed as percent of the total cells.

**RNA Extraction and RT-PCR array for autophagy genes**

To assess the expression of autophagy-related genes, the rat Autophagy RT² Profiler™ PCR Array kit (Qiagen SABiosciences, Frederick, MD) was used, which allows to study the expression of 84 key genes involved in autophagy. The assay was performed according to the specifications of the manufacturer. PC12-615 cells (8x10^5^ cells/60-mm dishes) were treated with NGF (10ng/ml) for 6h. After treatments, total RNA was extracted by using the RNeasy Mini Kit (Qiagen, Dusseldorf, Germany) and then incubated with RNase-free DNase (Qiagen) for 5 min at 42°C to eliminate genomic DNA contaminants. After measuring RNA concentration and purity, RNA (0.5 ng) was converted to cDNA using RT2 First Strand Kit for 20 min (15 min at 42°C; 5 min at 95°C). Real time PCR was performed with RT² SYBR Green ROX qPCR Mastermix using the Real-Time PCR System 7500 Fast (Applied Biosystem, Life Technologies) according to the manufacturer’s instructions. Quantification of RNA was accomplished by using the ΔΔC_t_ method (fold changes in Ct values of all genes). Relative amounts of mRNA for genes of interest were normalized to the housekeeping gene GAPDH.

**siRNAs sequences for Ambra1, Atg9b and Atg12**

siRNAs for Ambra1, Atg9b and Atg12 were synthesized by PRIMM s.r.l. (Milano, Italy) using the following sequences:

*A9b 1-1 5'-UCACCAAGAUCUACAGCUAUU-3'*

*A9b 1-2 5'-UAGCUGUAGAUCUUGGUGAUU-3'*

*A9b 2-1 5'-CCAAAGGAUUUCCAGGAGAUU-3'*

*A9b 2-2 5'-UCUCCUGGAAAUCCUUUGGUU-3'*

*A12 1-1 5'-CGGAGCAGUUGUUUAUUUAUU-3'*

*A12 1-2 5'-UAAAUAAACAACUGCUCCGUU-3'*

*A12 2-1 5'-AGACACUCCCAUAAUGAAAUU-3'*

*A12 2-2 5'-UUUCAUUAUGGGAGUGUCUUU-3'*

*A1 1-1 5'-AGGAAAUGCUCAACAAUAAUU-3'*

*A1 1-2 5'-UUAUUGUUGAGCAUUUCCUUU-3'*

*A1 2-1 5'-GGAUAAGACUCGAUGGAUGUU-3'*

*A1 2-2 5'-CAUCCAUCGAGUCUUAUCCUU-3'*

*SCR1 5'-GUCGAGAUAGGUGACAUAGUU-3'*

*SCR2 5'-CTATGTCACCTATCTCGACUU-3'*

**MDC staining for detection of acidic vacuoles**

Cells (7x10^4^ cells/well) were plated in 6-well plates (EuroClone) precoated with poly-L-lysine (0.1 mg/ml). After specific treatments, acidic vacuoles were assessed by adding 50 μM of MDC (Sigma) during the last 10 min of incubation. Cells were then washed with PBS, detached in PBS containing 0.1% TritonX-100, 10mM Tris HCl pH 8.00 and immediately analyzed using the Varian Cary Eclipse Fluorescence Spectrophotometer (Agilent Technologies). Excitation wavelength = 380 nm; emission wavelength = 460 nm.
